# Role of Patatin-Like Phospholipase Domain-Containing 3 on Lipid-Induced Hepatic Steatosis and Insulin Resistance in Rats

**DOI:** 10.1002/hep.26170

**Published:** 2013-01-25

**Authors:** Naoki Kumashiro, Toru Yoshimura, Jennifer L Cantley, Sachin K Majumdar, Fitsum Guebre-Egziabher, Romy Kursawe, Daniel F Vatner, Ioana Fat, Mario Kahn, Derek M Erion, Xian-Man Zhang, Dongyan Zhang, Vara Prasad Manchem, Sanjay Bhanot, Glenn S Gerhard, Kitt F Petersen, Gary W Cline, Varman T Samuel, Gerald I Shulman

**Affiliations:** 1 Howard Hughes Medical Institute, Yale University, School of MedicineNew Haven, CT; 2 Department of Internal Medicine, Yale University School of MedicineNew Haven, CT; 3 Department of Pediatrics, Yale University School of MedicineNew Haven, CT; 4 Department of Cellular & Molecular Physiology, Yale University School of MedicineNew Haven, CT; 5 ISIS PharmaceuticalsCarlsbad, CA; 6 Weis Center for Research, Geisinger ClinicDanville, PA; 7 Veterans Affairs Medical CenterWest Haven CT

## Abstract

Genome-wide array studies have associated the patatin-like phospholipase domain-containing 3 (PNPLA3) gene polymorphisms with hepatic steatosis. However, it is unclear whether PNPLA3 functions as a lipase or a lipogenic enzyme and whether PNPLA3 is involved in the pathogenesis of hepatic insulin resistance. To address these questions we treated high-fat-fed rats with specific antisense oligonucleotides to decrease hepatic and adipose pnpla3 expression. Reducing pnpla3 expression prevented hepatic steatosis, which could be attributed to decreased fatty acid esterification measured by the incorporation of [U-^13^C]-palmitate into hepatic triglyceride. While the precursors for phosphatidic acid (PA) (long-chain fatty acyl-CoAs and lysophosphatidic acid [LPA]) were not decreased, we did observe an ∼20% reduction in the hepatic PA content, ∼35% reduction in the PA/LPA ratio, and ∼60%-70% reduction in transacylation activity at the level of acyl-CoA:1-acylglycerol-sn-3-phosphate acyltransferase. These changes were associated with an ∼50% reduction in hepatic diacylglycerol (DAG) content, an ∼80% reduction in hepatic protein kinase Cε activation, and increased hepatic insulin sensitivity, as reflected by a 2-fold greater suppression of endogenous glucose production during the hyperinsulinemic-euglycemic clamp. Finally, in humans, hepatic PNPLA3 messenger RNA (mRNA) expression was strongly correlated with hepatic triglyceride and DAG content, supporting a potential lipogenic role of PNPLA3 in humans. *Conclusion:* PNPLA3 may function primarily in a lipogenic capacity and inhibition of PNPLA3 may be a novel therapeutic approach for treatment of nonalcoholic fatty liver disease-associated hepatic insulin resistance. ((Hepatology 2013;57:1763-1772))

Nonalcoholic fatty liver disease (NAFLD) is associated with hepatic insulin resistance, a major factor in the pathogenesis of type 2 diabetes (T2D) and the metabolic syndrome.^1^, ^2^ While patients carrying the I148M polymorphism in the patatin-like phospholipase domain-containing 3 (PNPLA3, also known as adiponutrin or calcium-independent phospholipase A_2_-epsilon) gene are prone to developing hepatic steatosis, the mechanism by which this occurs remains unknown.^3^–^9^ PNPLA3 has been reported to possess both triacylglycerol lipase and acylglycerol transacylase activities, specifically lysophosphatidic acid (LPA) acyltransferase activity,^10^*in vitro*.^10^–^14^ Some studies suggested a loss of lipase function by I148M genetic variant,^11^–^13^ but a recent report suggested I148M genetic variant causes a gain of lipogenic function.^10^ Thus, it has been unclear whether PNPLA3 functions as a lipase or lipogenic enzyme *in vivo*. To date, four reports assessed the physiological function of PNPLA3 using a gain- or loss-of-function approach in mice.^11^, ^15^–^17^ Evidence against PNPLA3 having any lipase activity was the absence of hepatic steatosis in pnpla3 knockout mice^16^, ^17^ and the observation that hepatic pnpla3 overexpression in mice did not reduce hepatic lipid content.^11^ In contrast, consistent with PNPLA3 functioning in a lipogenic capacity, Qiao et al.^15^ reported that hepatic pnpla3 knockdown using small interfering RNA (siRNA) in *ob/ob* and *db/db* mice had a slight tendency toward a decrease in hepatic triglyceride content associated with improved glucose tolerance in *db/db* mice. In addition, two recent human studies suggested an association of the PNPLA3 I148M variant with insulin resistance and hepatic steatosis,^8^, ^18^ in contrast to previous studies.^3^, ^5^–^7^ Therefore, PNPLA3 may play a lipogenic role and affect glucose tolerance, but the mechanisms of how PNPLA3 regulates hepatic lipid and glucose metabolism *in vivo* remain unclear.

To explore this question we knocked down hepatic and adipose pnpla3 expression with specific antisense oligonucleotides (ASOs) in rats and quantified hepatic lipogenesis using a novel stable isotope approach and assessed insulin-stimulated hepatic and peripheral glucose metabolism by hyperinsulinemic-euglycemic clamp studies in combination with stable and radiolabeled isotopes to assess insulin action in liver, muscle, and adipose tissue. One of the advantages of using this ASO approach is that the relative acute effects of reduced pnpla3 expression are examined in adult rats, thus avoiding any chronic adaptations that may occur in gene knockout mouse models. Furthermore, hepatic glucose metabolism in rats more closely resembles hepatic glucose metabolism in humans than mice. In addition, to examine whether our findings would translate to humans we also examined the relationship between hepatic PNPLA3 expression and hepatic triglyceride and diacylglycerol (DAG) content in liver biopsies that were obtained from obese patients with NAFLD undergoing gastric bypass surgery.

## Materials and Methods

### Animals

Male Sprague-Dawley rats (160-180 g) were obtained from Charles River Laboratories (Wilmington, MA) and given at least 3 days to acclimate before any studies. Rats were housed on a 12:12 hr light/dark cycle and received food and water *ad libitum*. Chow consisted of a regular rodent chow (60% carbohydrate, 10% fat, 30% protein calories) and a high-fat diet (Dyets 112245: 26% carbohydrate, 59% fat, 15% protein calories; Dyets, Bethlehem, PA). Body weight was monitored twice weekly. ASOs were injected intraperitoneally at a dose of 75 mg/kg per week for 4-5 weeks. Rats underwent the placement of jugular venous (for blood sampling) and carotid artery (for infusion) catheters ∼10 days before the infusion studies. They recovered their presurgical weights by 5-7 days after the operation. All procedures were approved by the Institutional Animal Care and Use Committee of Yale University School of Medicine.

### Hepatic Lipid Metabolites Assay

Hepatic triglyceride content was determined by using a triglyceride assay kit (Genzyme Diagnostics, PE, Canada) and a method adapted from Storlien et al.^19^ The extraction, purification, and assessment of medium, long-chain, very long-chain fatty acyl-CoAs, DAGs, phosphatidic acid (PA), and LPA from liver by liquid chromatography-mass spectrometry / mass spectrometry (LC-MS/MS) have been described.^20^–^23^ DAG fractionation into the membrane and the cytosolic lipid droplet compartments was performed as reported.^24^ Detailed methods for experiments are provided in the Supporting Methods.

### Reverse-Transcription Polymerase Chain Reaction (RT-PCR)

Total RNA was extracted from ∼15 mg liver or ∼80 mg epididymal adipose tissue using RNeasy mini kit (Qiagen, Valencia, CA). RNA was reverse-transcribed into complementary DNA (cDNA) with the use of M-MuLV Reverse Transcriptase (New England Biolabs, Ipswich, MA). The abundance of transcripts was assessed by real-time PCR on an Applied Biosystems 7500 Fast Real-Time PCR System (Applied Biosystems, Carlsbad, CA) with a SYBR Green detection system (Stratagene, La Jolla, CA). The expression data for each gene of interest were normalized for the efficiency of amplification with TATA box binding protein messenger RNA (mRNA) as the invariant control, as determined by a standard curve.^25^ Primer sequences are shown in Supporting Table 4.

### Western Blotting

Proteins were extracted using ∼100 mg liver. Akt, phosphorylated Akt, and pnpla3 were detected with whole cell lysates. Membrane translocation for protein kinase Cε (PKCε) was performed as described.^26^, ^27^ Detailed methods are provided in the Supporting Methods.

### Intraperitoneal Glucose Tolerance Test

After 3 weeks of ASOs treatment and ∼10 days before the studies, rats underwent the placement of jugular venous catheters. ASOs injection was continued twice weekly, rats were fasted overnight, and injected intraperitoneally with 20% dextrose (1.0 g/kg). Blood was taken from the venous line at the indicated time in the Results. Plasma glucose and insulin were subsequently measured as described in the Supporting Methods.

### Hyperinsulinemic-Euglycemic Clamp Studies

Hyperinsulinemic-euglycemic clamp studies were performed as described.^28^, ^29^ Full details are provided in the Supporting Methods.

### *In Vivo De Novo* Lipogenesis Assay

The liver or plasma samples for *de novo* lipogenesis assay, whole body lipolysis assay, and fatty acid esterification assay were taken from the same rats. Rats were treated with a high-fat diet and ASOs for 4 weeks, then 20 mL/kg of 99% deuterium oxide (D_2_O) (Cambridge Isotope Laboratories, Andover, MA) with 0.9% NaCl was injected intraperitoneally and maintained with 5% D_2_O-enriched drinking water *ad libitum* for 3 days. Rats were overnight fasted, then, 1 mg/mL U-^13^C palmitate (potassium palmitate 98 atom % D, Cambridge Isotope Laboratories), 5% bovine serum albumin (BSA) (cell culture tested, low endotoxin, fatty acid free, Sigma-Aldrich, St. Louis, MO), 100 mM glycerol-1,1,2,3,3-d5 (98 atom % D, Sigma-Aldrich) in 0.9% saline were infused at 75 μL/(kg-min) for 2.5 hours. After 2.5 hours, blood samples were obtained, then rats were anesthetized with sodium pentobarbital injection (75 mg/kg), and tissues were taken within 3 minutes, frozen immediately using cooled aluminum tongs in liquid N_2_, and stored at −80°C for the subsequent analysis.

Details for sample process and calculation are described in the Supporting Methods. *De novo* lipogenesis (%), newly synthesized palmitate in the hepatic triglyceride-palmitate, was calculated as described^30^ based on the incorporation of ^2^H from ^2^H_2_O onto newly synthesized palmitate molecules.

### In Vivo *Whole Body Lipolysis Assay*

Blood samples were obtained as described in the *In vivo de novo* lipogenesis assay section. Plasmas were used for lipolysis assay assessed by glycerol turnover.^31^ Details are described in the Supporting Methods.

### In Vivo *Fatty Acid Esterification Assay*

Liver samples were obtained as described in the *in vivo de novo* lipogenesis assay section.

Hepatic triglyceride-palmitate was extracted and analyzed for isotope enrichment as described in the *In vivo de novo* lipogenesis assay section. Mass isotopomer abundances were analyzed by selected ion monitoring with the atom percentage of enrichment (APE) of M_16_ (liver triglyceride-palmitate M_16_ APE) calculated from ions m/z 287 (M_16_) and 281 (M_0_).

The extraction procedure for hepatic palmitoyl-coenzyme A (palmitoyl-CoA) was performed as well as acyl-CoAs as described previously.^32^ Approximately 100 mg of frozen ground liver tissue was homogenized. Acyl-CoAs were purified using Oligonucleotide Purification Cartridges (Applied Biosystems, Foster City, CA) and eluted with 60% acetonitrile. The lipid extract was analyzed with an API 3000 LC-MS/MS system (AB Sciex, Framingham, MA), in negative mode using a turbo ion spray source in conjunction to a Shimadzu Prominence HPLC System (Shimadzu America, Columbia, MA). Palmitoyl-CoA M_16_ enrichment was calculated from multiple reaction mode (MRM) 501.9/924.5 (M_0_) and 509.9/940.5 (M_16_).

Finally, percent newly esterified triglyceride content was calculated with the equation: (% newly esterified triglyceride content) = (liver triglyceride-palmitate M_16_ APE) / (liver palmitoyl CoA M_16_ APE) × 100.

### LPA Acyltransferase Activity Assay

Total cell lysate proteins were extracted from 50 mg flash-frozen liver tissues from the same rats used for assessment of hepatic pnpla3 knockdown and lipid content as shown in the Supporting Materials and Methods. Twenty μg protein was incubated with 200 μM LPA (1-oleoyl-2-hydroxy-sn-glycero-3-phosphate, Avanti Polar Lipids, Alabaster, AL), 60 μM oleoyl CoA (Sigma-Aldrich), and 20 μM ^14^C oleoyl CoA or ^14^C palmitoyl CoA (PerkinElmer, San Jose, CA) in 200 μL of 50 mM Tris-HCl pH 7.4 solution at 37°C for 10 minutes. Then, 1.2 mL Folch solution (chloroform:methanol 2:1 vol/vol) and 400 μL of 2% phosphoric acid was added on ice. Samples were vortexed and centrifuged at 3,500 rpm for 10 minutes. The upper phase was discarded and bottom phase was dried under N_2_ and reconstituted in 60 μL chloroform. Thirty-μL samples (lipids) were spotted onto TLC plates (Thin Layer Chromatography Plates, Silica Gel 60 A, GE Healthcare Life Sciences, Piscataway, NJ) and separated in chloroform:methanol:acetone:glacial acetic acid:water (50:10:20:15:5, vol/vol) as the solvent system. Spots comigrating with phosphatidic acid standard (18:1 PA or 16:0-18:1 PA, Avanti Polar Lipids) were scraped and quantified by scintillation counter.

### Study Population

All patients who were enrolled in the Bariatric Surgery Program of the Geisinger Center for Nutrition and Weight Management between October 2004 and October 2010 were offered the opportunity to participate in this study and some others.^26^, ^33^ Over 90% of patients consented to participate. Patients who were previously diagnosed with diabetes or any other diseases were excluded from this study. Patients underwent a preoperative assessment and preparation program of monthly visits, during which time a comprehensive set of clinical and laboratory measures were obtained. The protocol was approved by the Institutional Review Boards of the Geisinger Clinic and Yale University, and all participants provided written informed consent.

### Liver Biopsies

During the bariatric surgery a wedge biopsy (250-300 mg) was obtained from the right lobe of the liver 10 cm to the left of the falciform ligament and flash-frozen in liquid nitrogen for subsequent analysis. The remainder was divided for routine histology. NAFLD was diagnosed with standard histological criteria.^34^

### Statistical Analysis

Linear regression analysis of the data was performed using Graph-Pad Prism 5.0. Data were compared using Student’s unpaired *t* test or analysis of variance (ANOVA) with the Tukey’s *post-hoc* test between two groups or more than two groups, respectively. All data are expressed as mean ± standard error of the mean (SEM) unless otherwise indicated. *P* < 0.05 was considered significant.

## Results

### Pnpla3 knockdown decreased hepatic DAG content in high-fat-fed (HFF) rats

**Fig 1:**
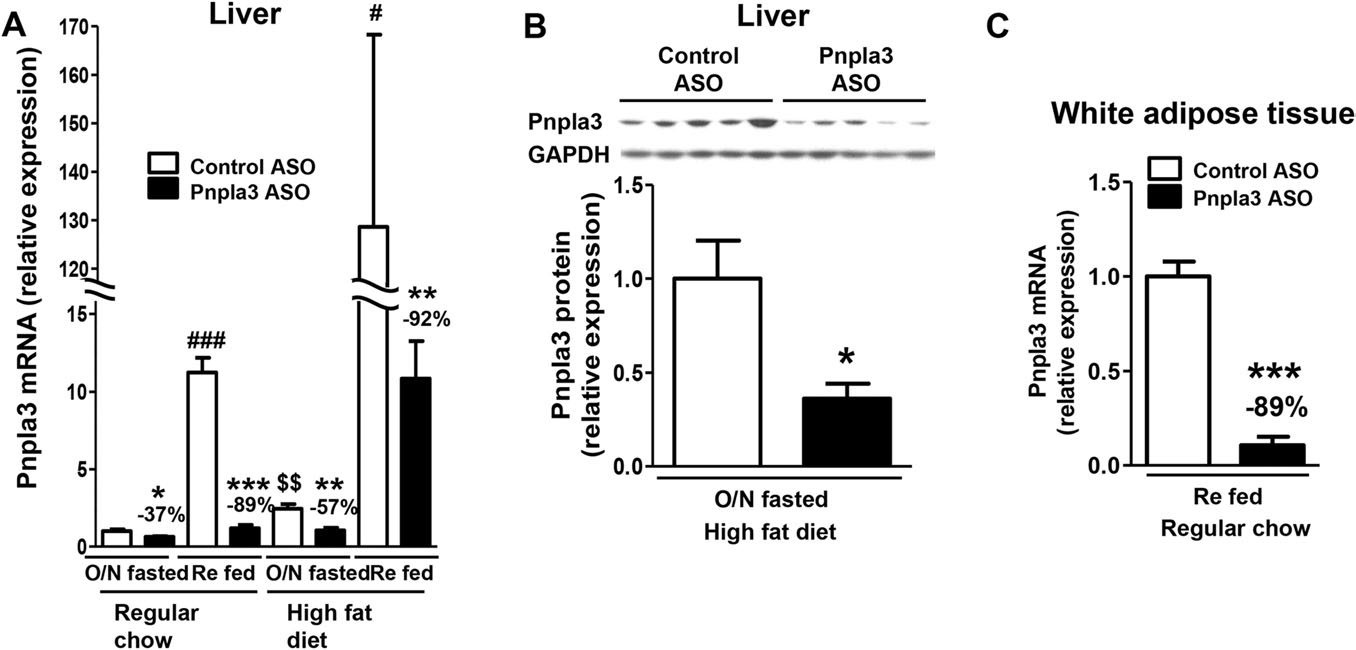
Pnpla3 ASO suppressed pnpla3 expression in liver and white adipose tissue. (A) Hepatic pnpla3 mRNA expression in overnight fasted (O/N fasted) or 5 hours refed condition in regular chow or HFF rats (n = 4-9 per group). The reduction percent in pnpla3 ASO rats compared with control ASO-treated rats in the same condition is shown. *P < 0.05, **P < 0.01, ***P < 0.001 compared with control ASO rats in the same condition, #*P* < 0.05, ###*P* < 0.001 compared between O/N fasted and refed condition in control ASO rats treated with the same diet. $$*P* < 0.01 compared between regular chow and high-fat diet condition in O/N fasted control ASO-treated rats. (B) Hepatic pnpla3 protein expressions in O/N fasted HFF rats (n = 5-6 per group). *P < 0.05 compared with control ASO rats. (C) Pnpla3 mRNA expression in epididymal adipose tissue in refed regular chow fed rats (n = 4 per group). ***P < 0.001 compared with control ASO rats. All data are expressed as mean ± SEM.

To assess the physiological role of PNPLA3 on lipid-induced hepatic steatosis and insulin resistance, we fed rats a high-fat diet and decreased hepatic and adipose pnpla3 expression by pnpla3 ASO treatment for 1 month. The advantage of this approach is that ASOs effectively silences gene expression primarily in liver and white adipose tissue,^29^, ^35^ where pnpla3 has been reported to express predominantly.^36^, ^37^ ASOs also avoid any compensatory developmental effects associated with gene-knockout mouse models. As shown in Fig. 1A, pnpla3 ASO treatment decreased hepatic pnpla3 expression by ∼50% in the fasted state in regular chow-fed and HFF rats. Refeeding strongly induced pnpla3 gene expressions (∼10-fold compared with fasted condition), consistent with previous observations and the role of this enzyme in lipid synthesis.^36^–^39^ The effects of the pnpla3 ASO were more pronounced following refeeding, with an ∼90% decrease in pnpla3 expression in comparison to control ASO-treated rats. Pnpla3 protein expression was also significantly decreased by pnpla3 ASO (Fig. 1B). This decrease was similarly observed in adipose tissue (Fig. 1C). Pnpla3 expression level was much higher in the white adipose tissue than in the liver (Supporting Fig. 1). Finally, high-fat feeding per se also significantly increased hepatic pnpla3 gene expression compared with regular chow fed condition. Of note, pnpla3 protein was predominantly localized in the cytosol fraction in HFF overnight-fasted rat livers (Supporting Fig. 2).

**Fig 2:**
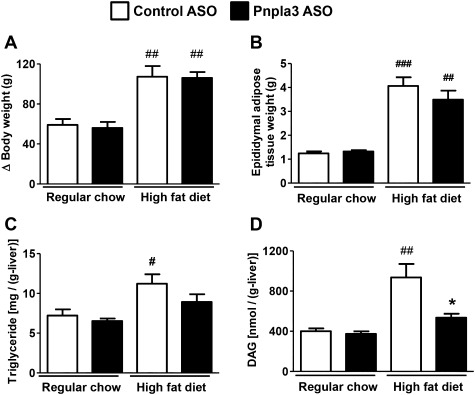
Pnpla3 ASO decreased hepatic lipid content in HFF rats. (A) Increase in body weight during the treatments in regular chow-fed and HFF rats treated with either a control or Pnpla3 ASO (n = 5-11 per group). (B-D) Epididymal adipose tissue weight, hepatic triglyceride content, hepatic DAG content, respectively, at sacrifice (n = 5-11 per group). #*P* < 0.05, ##*P* < 0.01, ###*P* < 0.001 compared to control ASO rats in regular chow fed condition. *P < 0.05 compared with control ASO rats in HFF condition. All data are expressed as mean ± SEM.

In comparison to regular chow fed rats, 1 month of high-fat diet feeding significantly increased adiposity and hepatic steatosis (Fig. 2). Pnpla3 ASO did not alter the development of adiposity, but decreased hepatic triglyceride content slightly (Fig. 2B,C; Supporting Fig. 3) and interestingly hepatic DAG content by ∼50% (Fig. 2D). Plasma lipid and adiponectin concentrations were not affected by pnpla3 ASO treatment (Supporting Table 1).

### Pnpla3 Knockdown Prevented Lipid-Induced Hepatic Insulin Resistance

**Fig 3:**
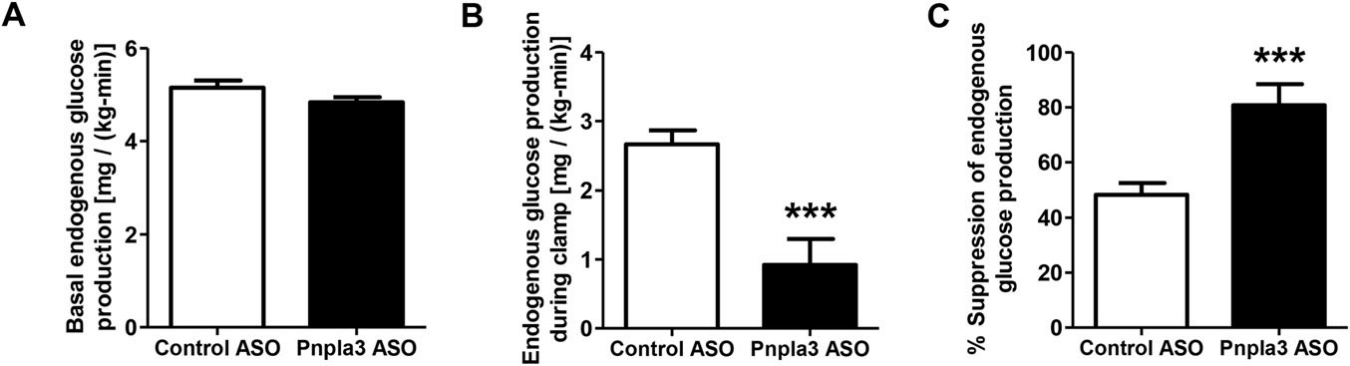
Pnpla3 ASO increased hepatic insulin sensitivity in HFF rats. (A) Basal endogenous glucose production (n = 9-10 per group). (B,C) Endogenous glucose production and percent suppression of endogenous glucose production during hyperinsulinemic-euglycemic clamps, respectively (n = 9-10 per group). ***P < 0.001 compared with control ASO-treated rats. All data are expressed as mean ± SEM.

**Fig 4:**
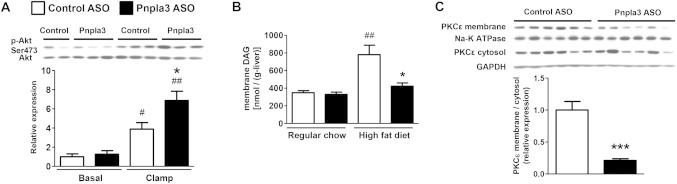
Pnpla3 ASO improved hepatic insulin signaling accompanied with a decrease in hepatic membrane DAG content and PKCε activation. (A) Akt phosphorylation (Ser473) assay in HFF rats (n = 4 for basal control ASO rats and n = 6 for the other groups), #*P* < 0.05 and ##*P* < 0.01 compared with control ASO rats in basal condition. *P < 0.05 compared with control ASO rats in clamp condition. (B) Membrane DAG content (n = 5-6 per group), ##*P* < 0.01 compared with control ASO rats in regular chow fed condition. *P < 0.05 compared with control ASO rats in HFF condition. (C) PKCε translocation assay in HFF rats (n = 6 per group), ***P < 0.001 compared with control ASO rats. All data are expressed as mean ± SEM.

To examine the impact of hepatic and adipose pnpla3 knockdown on glucose metabolism we performed intraperitoneal glucose tolerance tests in HFF rats. Plasma glucose concentrations 30 minutes after the glucose load were significantly lower in pnpla3 ASO rats than control ASO rats, with no difference in plasma insulin concentrations (Supporting Fig. 4). In order to assess tissue-specific changes in insulin sensitivity, we performed hyperinsulinemic [4 mU/(kg-min)]-euglycemic clamp studies in conjunction with stable and radiolabeled isotopes to assess insulin action in liver, muscle, and adipose tissue. Although there were no observable differences in insulin-stimulated peripheral glucose metabolism (Supporting Fig. 5) during the hyperinsulinemic-euglycemic clamp, insulin-mediated suppression of endogenous glucose production was ∼2-fold greater in pnpla3 ASO rats than control ASO-treated rats (Fig. 3). Thus, pnpla3 ASO treatment in HFF rats primarily improved hepatic insulin sensitivity. This protection from lipid-induced hepatic insulin resistance could be attributed to improvements in hepatic insulin signaling, as assessed by Akt phosphorylation at Ser473 (Fig. 4A). Akt phosphorylation at Thr308 was not changed significantly (Supporting Fig. 6). Previous studies have implicated DAG-mediated activation of PKCε as causing hepatic insulin resistance in NAFLD.^1^, ^26^, ^28^ Consistent with this mechanism in the pnpla3 ASO-treated rats, we observed an ∼50% reduction in hepatic membrane DAG content and PKCε activation (Fig. 4B,C). Although all membrane DAG species were lower in pnpla3 ASO-treated rats compared to control ASO-treated rats, the greatest reduction occurred in the (C18:2, C18:2), (C18:1, C18:2), and (C16, C18:2) DAG species (Supporting Table 2).

### Pnpla3 Knockdown Suppressed Hepatic Fatty Acid Esterification *In Vivo*

**Fig 5:**
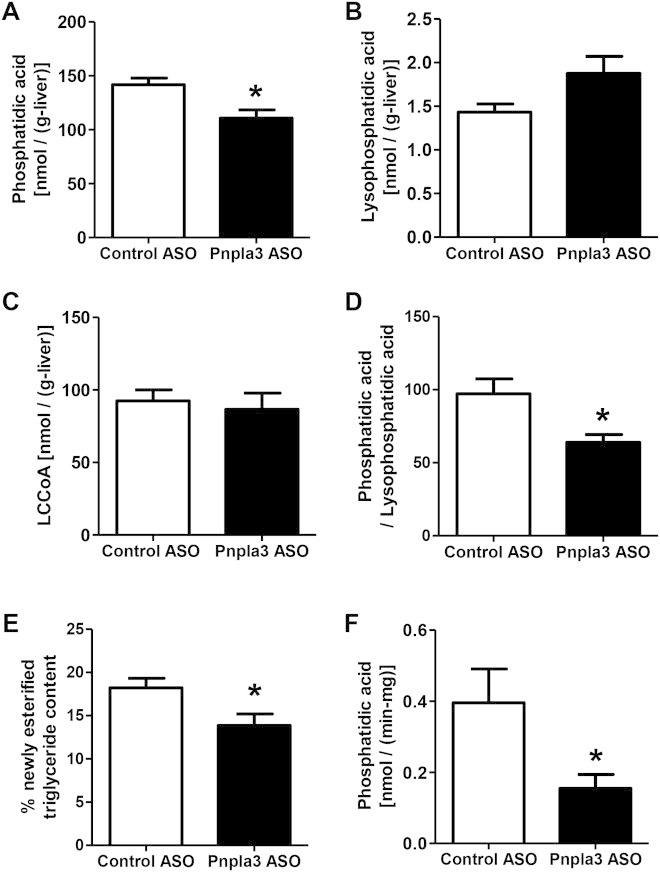
PNPLA3 ASO decreased hepatic fatty acid esterification in HFF rats. (A-C) Hepatic phosphatidic acid, lysophosphatidic acid, and long-chain fatty acyl-CoA (LCCoA) content, respectively (n = 6 per group). (D) Hepatic phosphatidic acid / lysophosphatidic acid ratio (n = 6 per group). (E) *In vivo* hepatic fatty acid esterification assay (n = 7 per group). (F) Lysophosphatidic acid acyltransferase activity assay (n = 6 per group). Protein samples were extracted from total liver lysate using flash-frozen livers, which are the same livers used for knockdown confirmation, lipid content, and PKCε assays in HFF overnight fasted condition, then incubated with ^14^C-palmitoyl CoA and lysophosphatidic acid. Produced ^14^C-labeled phosphatidic acid was measured with a scintillation counter. *P < 0.05 compared to control ASO rats. All data are expressed as mean ± SEM.

**Fig 6:**
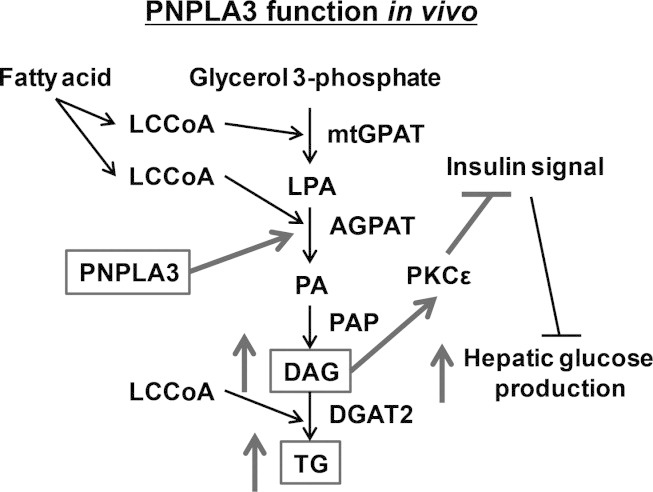
The lipogenic role of PNPLA3 on hepatic steatosis and hepatic insulin resistance *in vivo*. LCCoA, long-chain fatty acyl-coenzyme A; mtGPAT, mitochondrial acyl-CoA:glycerol-sn-3-phosphate acyltransferase; LPA, lysophosphatidic acid; AGPAT, acyl-CoA:1-acylglycerol-sn-3-phosphate acyltransferase; PA, phosphatidic acid; PAP, phosphatidic acid phosphatase; DAG, diacylglycerol; DGAT2; acyl-CoA:diacylglycerol acyltransferase 2; TG, triglyceride; PKCε, protein kinase Cε.

We next investigated the mechanism responsible for the prevention of lipid-induced hepatic steatosis by pnpla3 knockdown. First, we measured PA content, which is the precursor for DAGs. Parallel to hepatic DAG content, hepatic PA content was ∼20% lower in Pnpla3 ASO-treated rats compared to control ASO-treated rats (Fig. 5A). However, interestingly, the precursors for PA (long-chain fatty acyl-CoAs [LCCoAs] and LPA) were not decreased with Pnpla3 ASO treatment but LPA tended to increase (Fig. 5B,C) and there was a significant decrease (∼35%) in the PA/LPA ratio (Fig. 5D). We also assessed *in vivo* hepatic fatty acid esterification by measuring the incorporation of [U-^13^C]-palmitate into hepatic triglyceride. Pnpla3 ASO decreased the esterification of [U-^13^C]-palmitate into hepatic triglyceride by ∼25% (Fig. 5E). We assessed LPA acyltransferase activity using liver lysates, and we found that LPA acyltransferase activity was reduced ∼60%-70% by pnpla3 knockdown (Fig. 5F; Supporting Fig. 7A). These data suggest that PNPLA3 plays a lipogenic role in liver through fatty acid esterification primarily at the level of acyl-CoA:1-acylglycerol-sn-3-phosphate acyltransferase (AGPAT) (Fig. 6). Interestingly, the relative contribution of hepatic *de novo* fatty acid synthesis to hepatic triglyceride synthesis, assessed by the incorporation of ^2^H from ^2^H_2_O into triglyceride palmitate *in vivo*, was significantly increased in Pnpla3 ASO rats (Supporting Fig. 7B), suggesting a compensatory role of hepatic *de novo* fatty acid synthesis to hepatic triglyceride synthesis with reduced pnpla3 expression. Consistent with this relative increase in hepatic *de novo* fatty acid synthesis, we observed an increased expression of hepatic acetyl-CoA carboxylase 1 (ACC1) and fatty acid synthase (FAS) mRNA in Pnpla3 ASO-treated rats compared to control ASO-treated rats (Table 1). In contrast, whole body lipolysis, as assessed by glycerol turnover, was not changed by suppression of both hepatic and adipose pnpla3 expression (Supporting Fig. 7C). In addition, because the PNPLA3 genetic variant has been reported to be associated with morphological changes in adipocyte cell size,^40^ we measured adipocye cell size but found no difference in fat cell size between the groups. (Supporting Fig. 8).

**Table 1:** Lipid Metabolism Related Gene Expressions in Liver

		HFF Fasted		HFF Refed	
		Control ASO	Pnpla3 ASO	Control ASO	Pnpla3 ASO
Lipogenic genes	SREBP1c	1.00 ± 0.11	1.26 ± 0.32§	5.28 ± 0.74*	6.92 ± 1.34†
	ACC1	1.00 ± 0.06	1.33 ± 0.13	1.47 ± 0.95*	1.87 ± 0.12‡,§
	FAS	1.00 ± 0.21	1.14 ± 0.20∥	7.68 ± 1.42†	12.8 ± 1.54‡,§
	mGPAT	1.00 ± 0.16	1.03 ± 0.16	1.54 ± 0.19	1.76 ± 0.19*
	AGPAT1	1.00 ± 0.13	0.92 ± 0.10	0.95 ± 0.04	0.83 ± 0.07
	AGPAT2	1.00 ± 0.18	1.16 ± 0.17	1.18 ± 0.10	1.45 ± 0.14
	AGPAT3	1.00 ± 0.10	0.91 ± 0.08	0.76 ± 0.07	0.82 ± 0.05
	AGPAT4	1.00 ± 0.11	1.48 ± 0.30§	0.81 ± 0.08	1.20 ± 0.08
	AGPAT5	1.00 ± 0.07	1.10 ± 0.15	1.09 ± 0.06	1.13 ± 0.06
	AGPAT6	1.00 ± 0.12	1.43 ± 0.10*,¶	0.75 ± 0.06	0.79 ± 0.05
	AGPAT9	1.00 ± 0.11	0.94 ± 0.15¶	0.17 ± 0.05‡	0.10 ± 0.03‡
	PAP	1.00 ± 0.24	1.03 ± 0.15	0.93 ± 0.07	1.03 ± 0.07
	DGAT2	1.00 ± 0.07	1.08 ± 0.12	1.16 ± 0.05	1.50 ± 0.13†,§
Hydrolysis gene	ATGL	1.00 ± 0.09	1.05 ± 0.08¶	0.48 ± 0.03‡	0.50 ± 0.03‡
Lipid oxidation	PPARα	1.00 ± 0.16	0.93 ± 0.10	0.78 ± 0.08	0.91 ± 0.08
Genes	CPT1	1.00 ± 0.15	0.93 ± 0.11∥	0.47 ± 0.05‡	0.41 ± 0.03‡

Data are mean ± SEM.

* *P* < 0.05,

† *P* < 0.01,

‡ *P* < 0.001 compared with overnight fasted control ASO treated rats.

§ *P* < 0.05,

∥ *P* < 0.01,

¶ *P* < 0.001 compared with 5 hours refed control ASO treated rats. HFF, high-fat fed; SREBP, sterol regulatory element binding transcription factor; ACC, acetyl-CoA carboxylase; FAS, fatty acid synthase; mGPAT, mitochondrial acyl-CoA:glycerol-sn-3-phosphate acyltransferase; AGPAT, acyl-CoA:1-acylglycerol-sn-3-phosphate acyltransferase; AGPAT1, 2, 3, 4, 5, 6, and 9 are transcript variants of AGPAT; PAP, phosphatidic acid phosphatase; DGAT, acyl-CoA:diacylglycerol acyltransferase; ATGL, adipocyte triglyceride lipase; PPARα, peroxisome proliferator activated receptor alpha; CPT, carnitine palmitoyl transferase.

**Fig 7:**
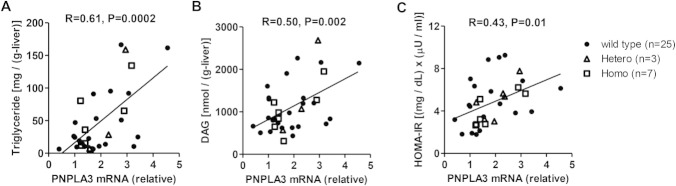
Hepatic PNPLA3 mRNA expression level positively correlated with hepatic triglyceride, DAG content, and insulin resistance in humans. (A,B) Correlation between hepatic triglyceride or DAG content and hepatic PNPLA3 mRNA expression in humans (n = 35). (C) Correlation between hepatic PNPLA3 mRNA expression and HOMA-IR (n = 35). Black dots are wildtype (148I) subjects, triangles are 148M variant heterozygous carriers, and squares are 148M variant homozygous carriers.

Finally, we assessed whether expression of PNPLA3 is altered in patients with NAFLD by assessing PNPLA3 gene expression in liver biopsies obtained from nondiabetic obese subjects with NAFLD (Supporting Table 3). As shown in Fig. 7A,B, there was a large variation in hepatic PNPLA3 mRNA expression level, and it was positively correlated with hepatic triglyceride and DAG content, supporting the hypothesis that PNPLA3 plays a lipogenic role in humans with NAFLD. The hepatic PNPLA3 expression level was also correlated with insulin resistance as assessed by homeostatic model assessment of insulin resistance index (HOMA-IR) (Fig. 7C). These correlations were not affected by the presence of polymorphisms in the PNPLA3 gene.

## Discussion

In order to examine the role of PNPLA3 in the regulation of hepatic lipid and glucose metabolism *in vivo*, we knocked down pnpla3 gene and protein expression using a pnpla3-specific ASO in rats. The advantage of this approach is that ASOs have inherent tissue specificity, effectively silencing gene expression in liver and white adipose tissue,^29^, ^35^ where pnpla3 is predominantly expressed,^36^, ^37^ and ASOs avoid any compensatory developmental effects associated with gene-knockout mouse models. We found that pnpla3 ASO treatment decreased hepatic DAG content and protected rats from lipid-induced hepatic insulin resistance, which could be attributed to decreased DAG-mediated PKCε activation.^1^, ^41^ In addition, we demonstrated that pnpla3 plays a lipogenic role through fatty acid esterification at the level of AGPAT *in vivo*.

Supporting a lipogenic role of PNPLA3, feeding increased pnpla3 gene expression similar to the other lipogenic genes such as ACC1 and FAS in contrast to the decreased lipolytic enzyme, adipocyte triglyceride lipase (ATGL, also known as PNPLA2) (Fig. 1; Table 1). As a lipogenic enzyme, PNPLA3 was reported to be transcriptionally regulated by sterol regulatory element binding transcription factor 1c (SREBP1c)^15^, ^37^ and carbohydrate response element-binding protein (ChREBP)^13^, ^42^*in vitro* using promoter binding assay, which suggests that PNPLA3 plays a lipogenic role through *de novo* fatty acid synthesis.^43^, ^44^ Surprisingly, we found that the relative contribution of *de novo* fatty acid synthesis to hepatic triglyceride synthesis was increased by pnpla3 knockdown *in vivo*, suggesting that PNPLA3 is not responsible for *de novo* fatty acid synthesis *in vivo*. Instead, pnpla3 knockdown decreased fatty acid esterification at the level of AGPAT based on the observed crossover between hepatic LPA and PA content and the decrease in LPA acyltransferase activity. This is consistent with recent *in vitro* studies showing that PNPLA3 promotes lipogenesis by converting LPA into PA^10^ and lipid accumulation into hepatocytes was observed in the presence of fatty acid but not observed with glucose.^13^ Taken together, these data suggest that PNPLA3 plays a lipogenic role by promoting fatty acid esterification at the level of AGPAT *in vivo*.

We also found that pnpla3 ASO-treated rats were protected from lipid-induced hepatic insulin resistance. These findings are consistent with previous observations demonstrating improved glucose tolerance in HFF pnpla3 knockout mice and in obese mice with decreased hepatic pnpla3 expression by siRNA injection, although no physiological or cellular mechanism was provided.^15^, ^17^ In this regard, we found that pnpla3 ASO-treated rats were primarily protected from lipid-induced hepatic insulin resistance without improvements in insulin-stimulated peripheral glucose metabolism. We went on to show that this protection from lipid-induced hepatic insulin resistance was associated with marked reductions in hepatic DAG content, decreased hepatic PKCε activity, and increased insulin-stimulated Akt phosphorylation, consistent with previous studies in humans and animals implicating a causal role of DAG-mediated PKCε activation in mediating hepatic insulin resistance.^1^ Finally, in order to examine if these results translate to humans, we found that hepatic PNPLA3 expression was positively correlated with hepatic DAG content and insulin resistance in humans.

A potential limitation of this study is the species difference of PNPLA3 between rats and humans. Rat pnpla3 is more abundant in adipose tissue than in liver and more abundant in cytosol than in membrane or lipid droplets, which is different from what has been reported in humans.^14^, ^37^ However, our findings demonstrating a strong positive correlation between hepatic PNPLA3 expressions and hepatic lipid content and whole body insulin resistance in humans are consistent with our results demonstrating protection of pnpla3 ASO-treated rats from lipid-induced hepatic insulin resistance. Recently, Pirazzi et al.^45^ proposed a model in which PNPLA3 is involved in very low-density lipoprotein (VLDL) secretion by overexpressing human PNPLA3 wild and mutant proteins into rat hepatoma cells. Although our present studies clearly demonstrated the lipogenic function of pnpla3 through LPA acyltransferase activity, the potential function on VLDL secretion should be examined in future studies. In addition, a recent study by Li et al.^46^ demonstrated the effects of human PNPLA3 148I and mutant 148M overexpression in mice liver. They found impaired hydrolysis in 148M overexpressed primary hepatocytes and unchanged acyltransferase activity in 148M overexpression mice, which may be due to species difference or differences in the enzymatic activity of PNPLA3 148M compared to native pnpla3.

Taken together, these data support the hypothesis that pnpla3 in rat liver primarily acts in a lipogenic role through fatty acid esterification at the level of LPA acyltransferase and that decreasing pnpla3 expression prevents lipid-induced whole body insulin resistance by preventing lipid-induced hepatic insulin resistance by decreasing hepatic DAG accumulation and activation of PKCε. These data also suggest that inhibition of PNPLA3 activity may represent a novel therapeutic approach for the treatment of NAFLD-associated hepatic insulin resistance.

## Abbreviations

ACC: acetyl-CoA carboxylase
AGPAT: acyl-CoA:1-acylglycerol-sn-3-phosphate acyltransferase
ALT: alanine aminotransferase; APE, atom percentage of enrichment
ASOs: antisense oligonucleotides; ATGL, adipocyte triglyceride lipase
DAG: diacylglycerol; FAS, fatty acid synthase; HFF, high-fat fed
HOMA-IR: homeostatic model assessment of insulin resistance index
LC-MS/MS: liquid chromatography-mass spectrometry / mass spectrometry; LCCoAs, long-chain fatty acyl-CoAs
LPA: lysophosphatidic acid; NAFLD, nonalcoholic fatty liver disease
PKCε: protein kinase Cε; PA, phosphatidic acid; PNPLA3, patatin-like phospholipase domain-containing 3
T2D: type 2 diabetes.
