# Supplementary figures and images for: Role of Patatin-Like Phospholipase Domain-Containing 3 on Lipid-Induced Hepatic Steatosis and Insulin Resistance in Rats
Source: Hepatology. 2013 Jan 25;57(5):1763–72. doi: 10.1002/hep.26170 (PMC3597437; doi:10.1002/hep.26170)

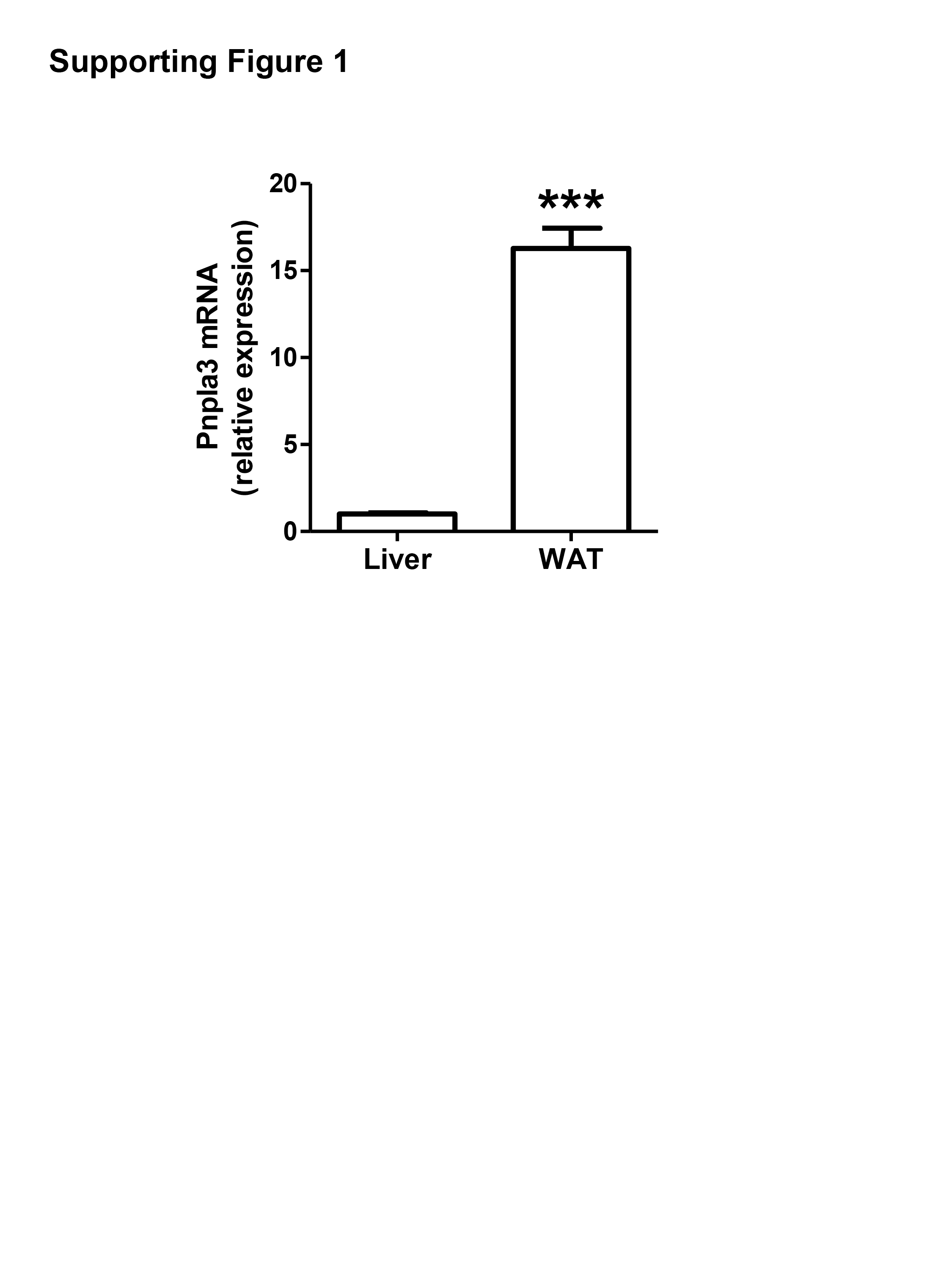

Supplement: Supplementary file 1 [file hep0057-1763-sd1.tif]

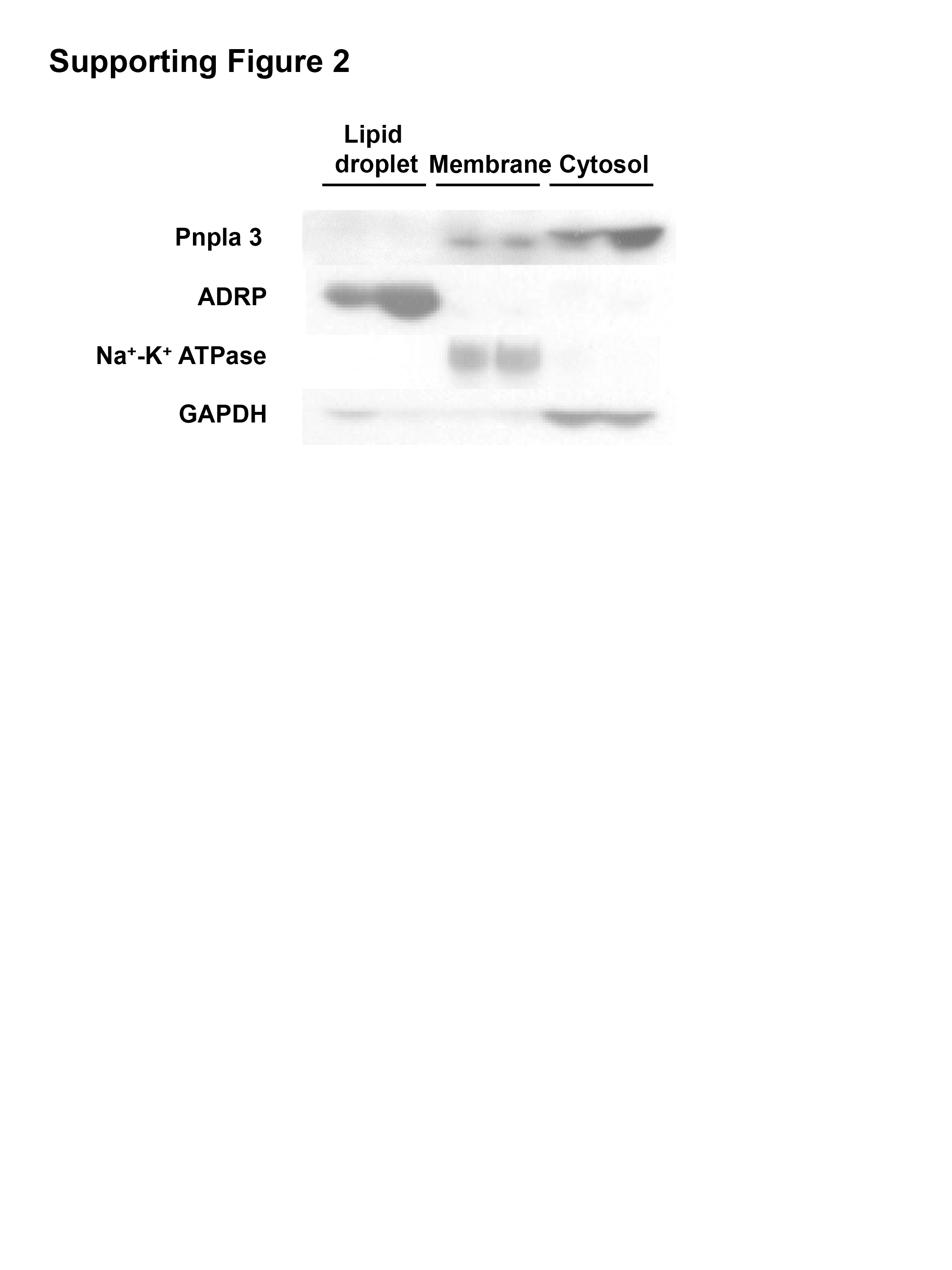

Supplement: Supplementary file 2 [file hep0057-1763-sd2.tif]

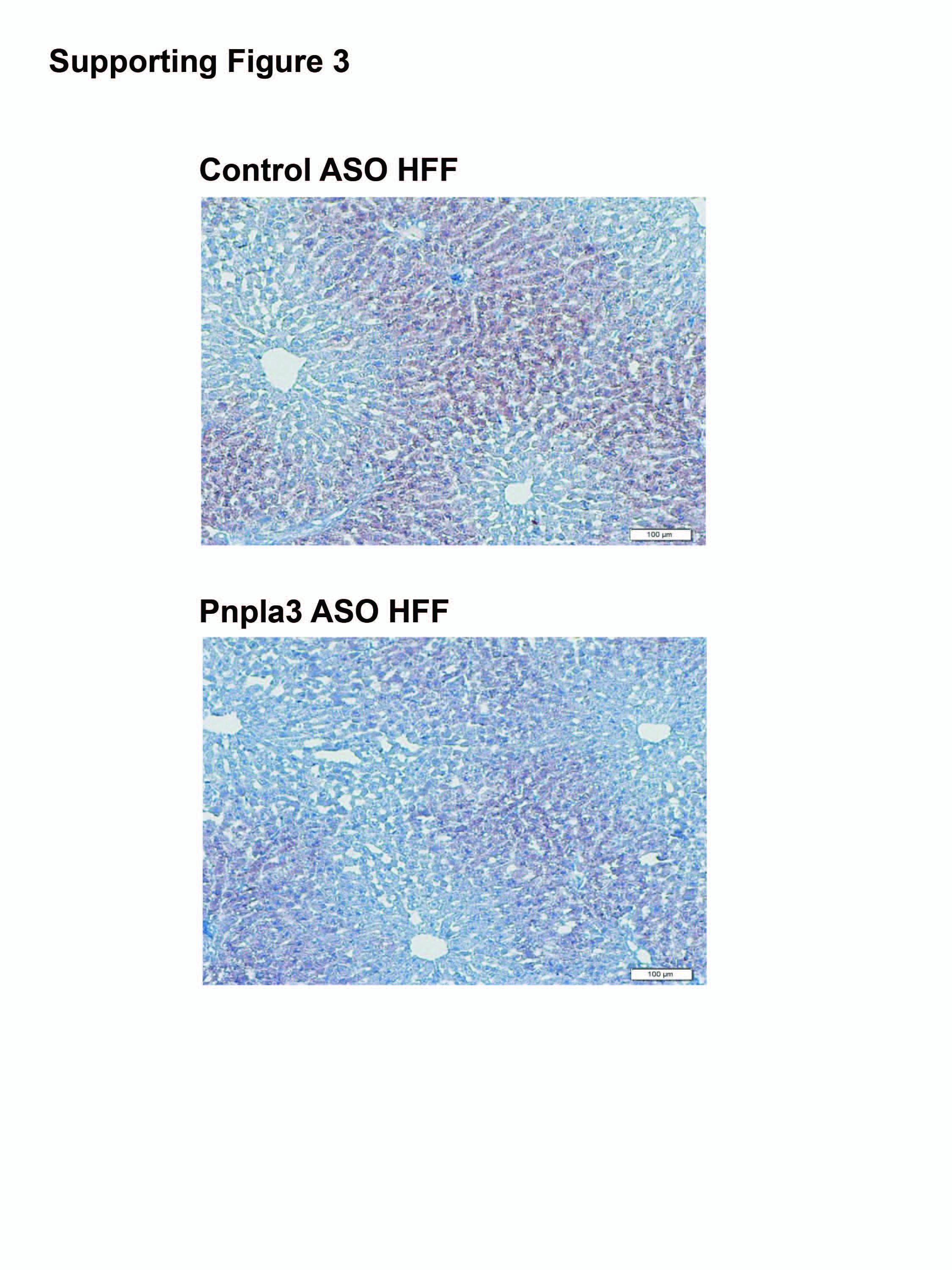

Supplement: Supplementary file 3 [file hep0057-1763-sd3.tif]

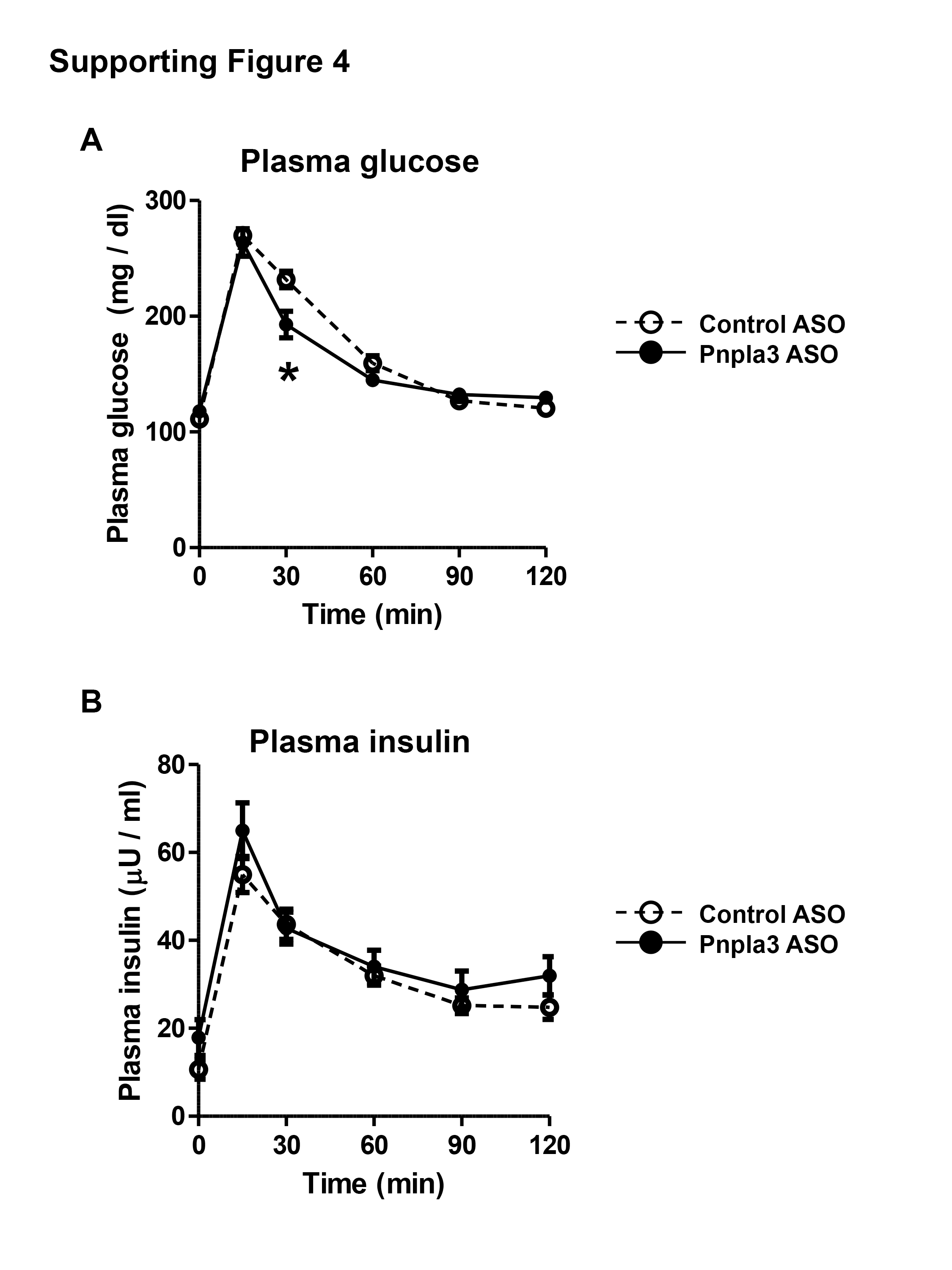

Supplement: Supplementary file 4 [file hep0057-1763-sd4.tif]

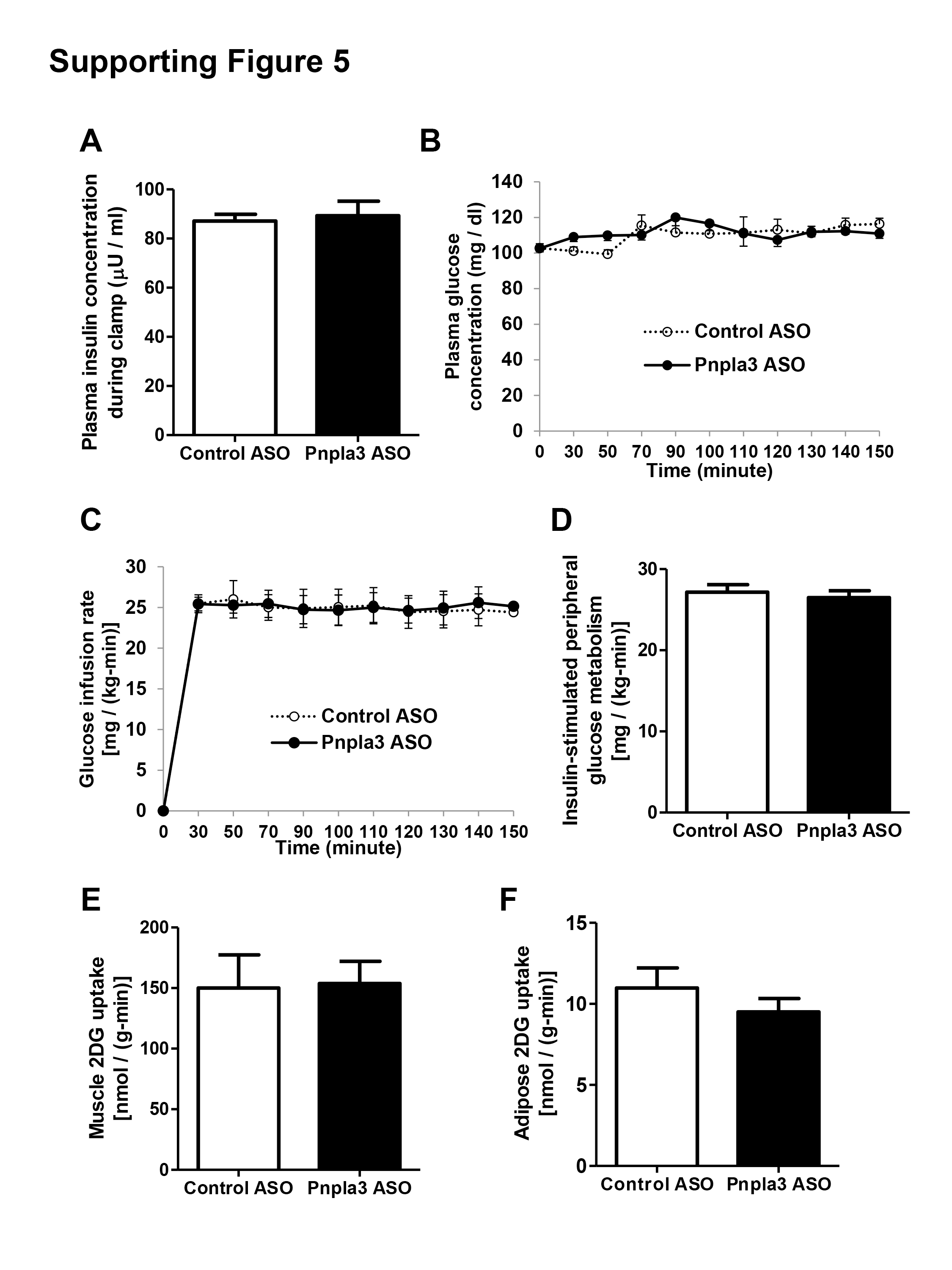

Supplement: Supplementary file 5 [file hep0057-1763-sd5.tif]

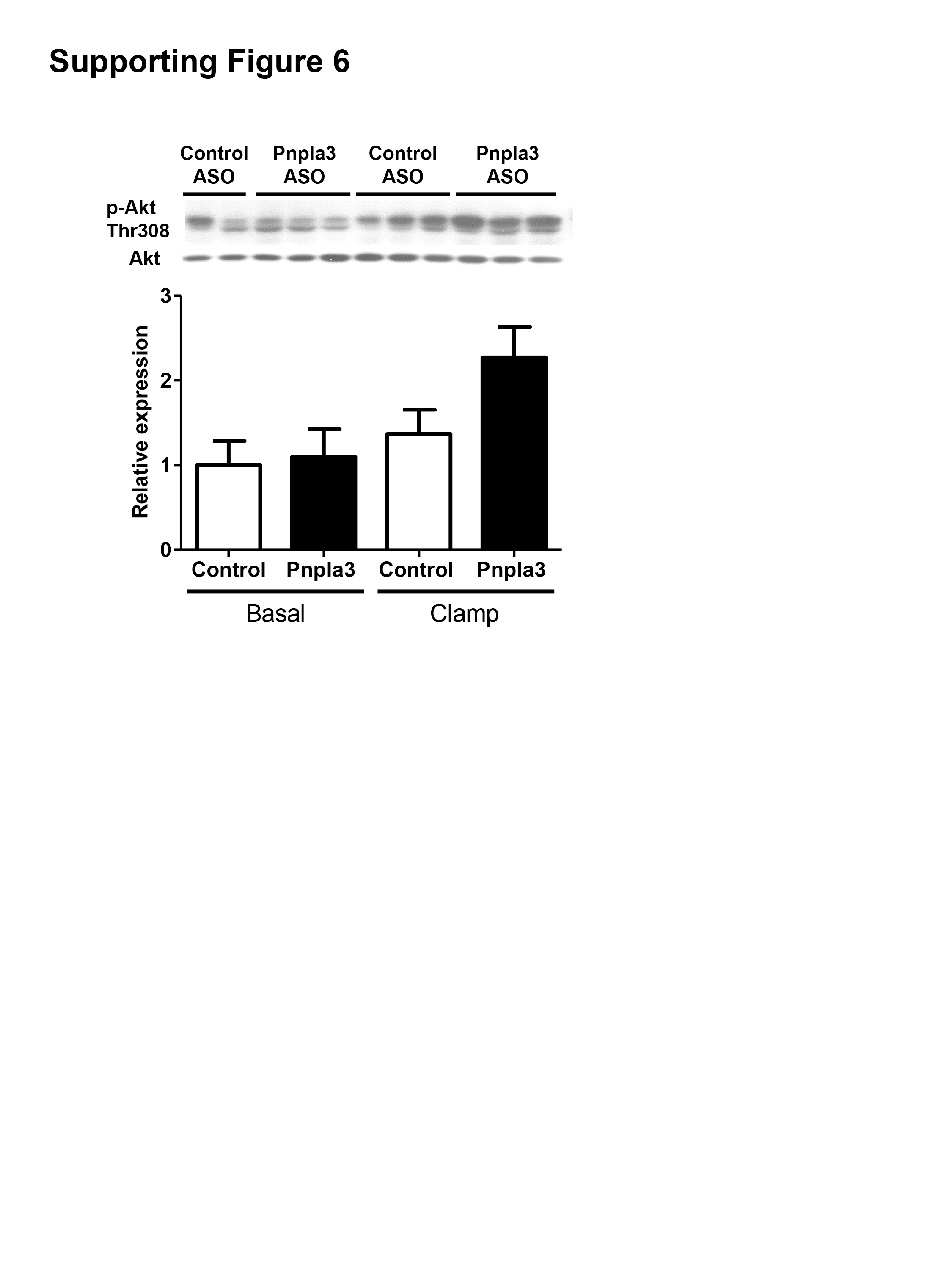

Supplement: Supplementary file 6 [file hep0057-1763-sd6.tif]

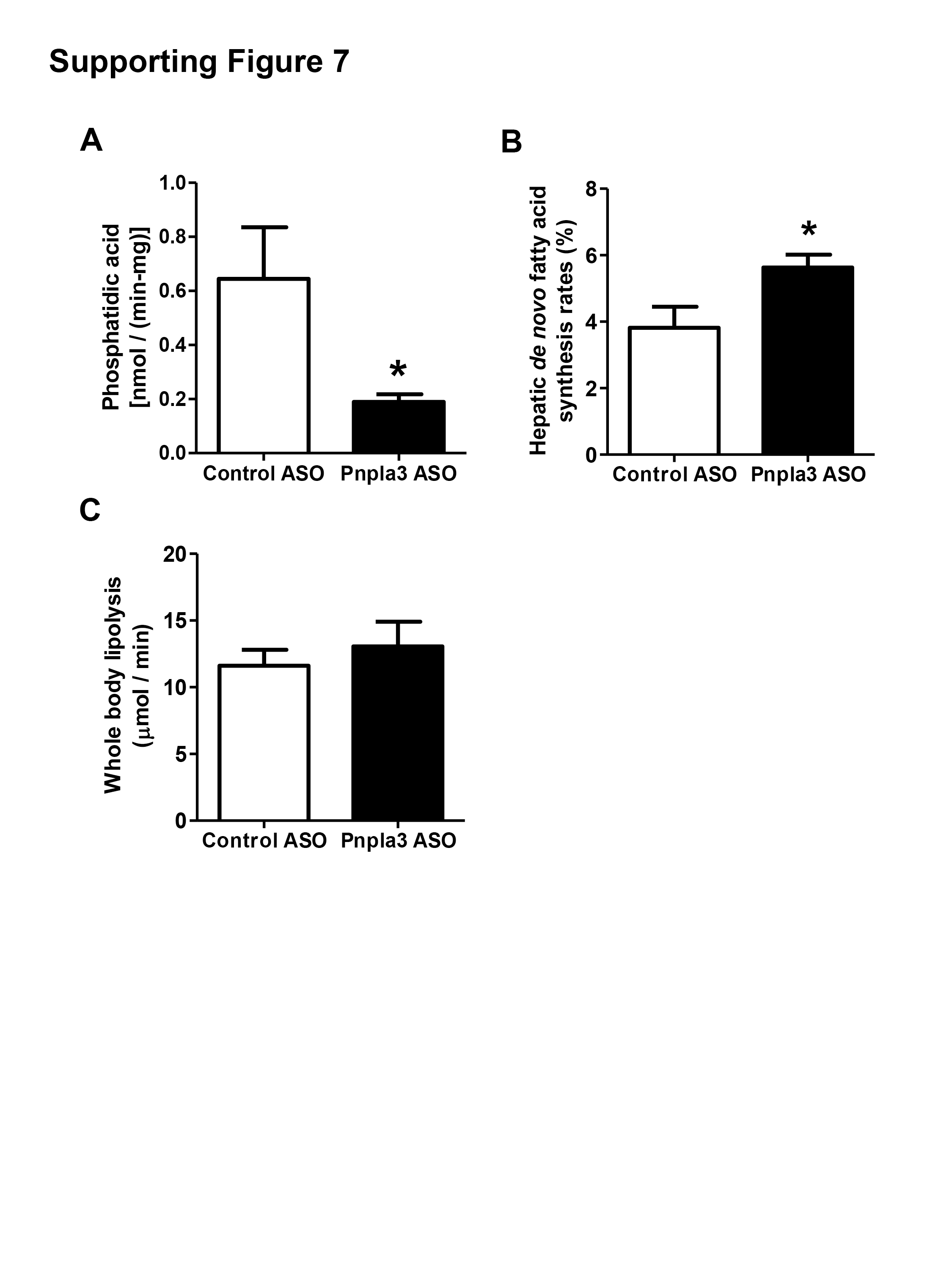

Supplement: Supplementary file 7 [file hep0057-1763-sd7.tif]

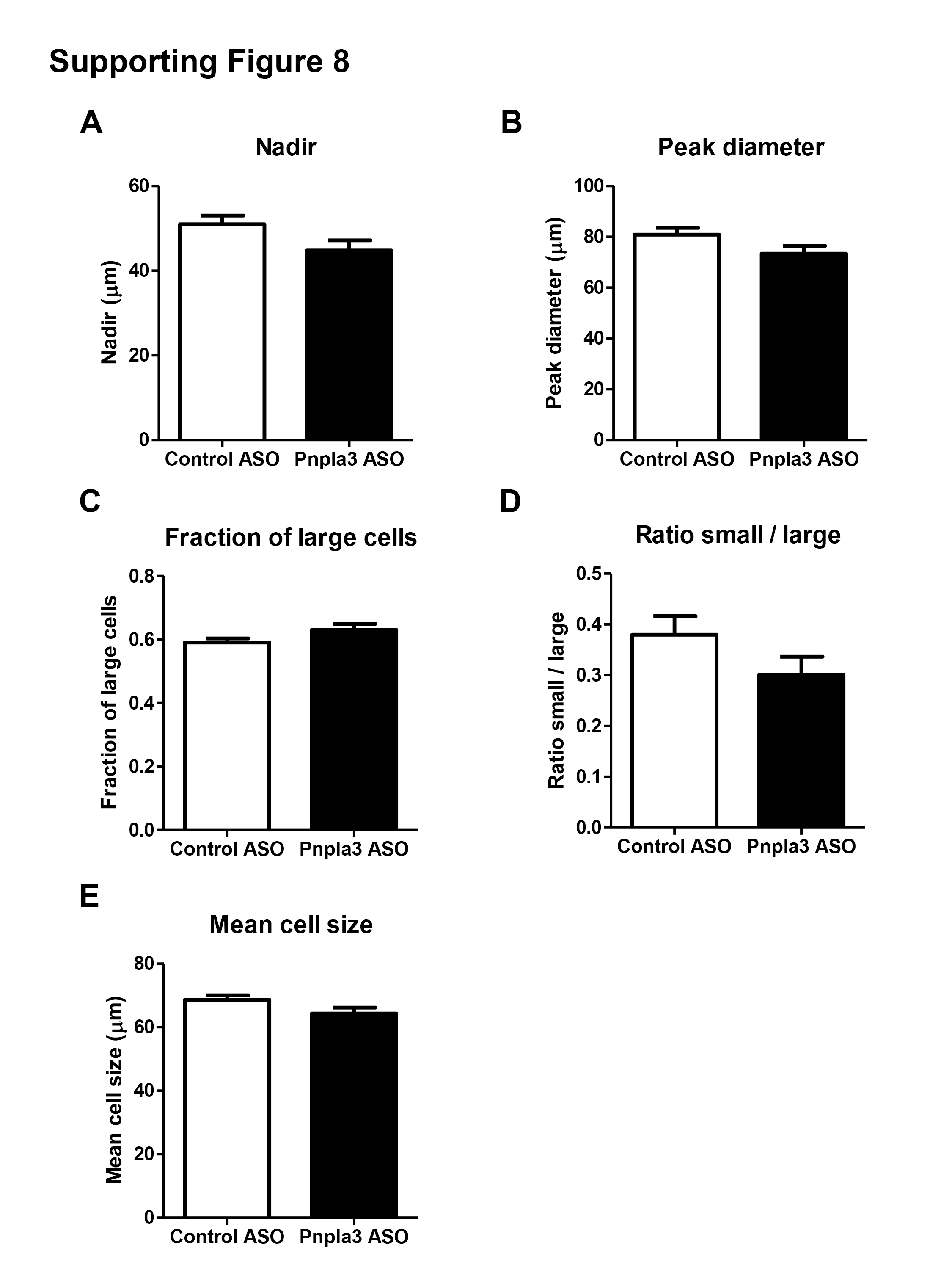

Supplement: Supplementary file 8 [file hep0057-1763-sd8.tif]
