# Supplementary material for: Role of Patatin-Like Phospholipase Domain-Containing 3 on Lipid-Induced Hepatic Steatosis and Insulin Resistance in Rats
Source: Hepatology. 2013 Jan 25;57(5):1763–72. doi: 10.1002/hep.26170 (PMC3597437; doi:10.1002/hep.26170)
Supplement: Supplementary file 9 [file hep0057-1763-sd9.doc]

**Supporting Information**

**The Role of Patatin-Like Phospholipase Domain-Containing 3 on**

**Lipid-induced Hepatic Steatosis and Insulin Resistance in Rats**

Naoki Kumashiro, Toru Yoshimura, Jennifer L Cantley, Sachin K Majumdar,

Fitsum Guebre-Egziabher, Romy Kursawe, Daniel F Vatner, Ioana Fat, Mario Kahn,

Derek M Erion, Xian-Man Zhang, Dongyan Zhang, Vara Prasad Manchem,

Sanjay Bhanot, Glenn S. Gerhard, Kitt F Petersen, Gary W Cline, Varman T Samuel, and Gerald I Shulman

**Inventory of Supporting Information**

I. Supporting Figures **on individual TIFF files**

Figure 1. Comparison of pnpla3 mRNA expression between liver and white

adipose tissue.

Figure 2. Pnpla3 cellular localization in the rat liver.

Figure 3. Representative pictures of oil red O staining.

Figure 4. Intraperitoneal glucose tolerance test in HFF condition.

Figure 5. Peripheral insulin sensitivity assessed by hyperinsulinemic-euglycemic

clamp.

Figure 6. Akt phosphorylation at Thr308.

Figure 7. Lysophosphatidic acid acyltransferase acitivity with oleoyl CoA,

*de novo* fatty acid synthesis in liver, and whole body lipolysis.

Figure 8. Adipocyte sizing results.

II. Supporting Tables **on individual Word documents**

Table 1. Plasma data.

Table 2. DAG species analysis.

Table 3. Characteristics of participants.

Table 4. Primer sequences.

III. Supporting Figure Legends

IV. Supporting Materials and Methods

V. Supporting References

**Supporting Figure Legends**

**Supporting Figure 1.** Comparison of pnpla3 mRNA expression between liver and white adipose tissue (WAT).

Samples were prepared with regular chow 5 hours refed control ASO treated rat tissues (n = 4 per group). The average of hepatic pnpla3 expression was set as 1. *** P<0.001 compared between tissues.

**Supporting Figure 2.** Pnpla3 cellular localization in the rat liver.

50 μg protein from high-fat fed control ASO treated overnight fasted liver was used for each fraction. Adipocyte differentiation-related protein (ADRP) is a lipid droplet marker, Na^+^-K^+^ ATPase is a membrane marker, and GAPDH is a cytosol marker. All the detections were done with the same blotted membrane by stripping and reprobing.

**Supporting Figure 3.** Representative pictures of oil red O staining.

Oil red O staining was done with high-fat fed refed rat livers.

**Supporting Figure 4.** Intraperitoneal glucose tolerance test in HFF condition.

(A,B) Plasma glucose and insulin concentration, respectively, at the indicated time after intraperitoneal glucose loading [1 g / (kg-BW)] in HFF rats (n = 7 - 9 per group). * P<0.05 compared between control and pnpla3 ASO treated rats by ANOVA with the Tukey’s post-hoc test.

**Supporting Figure 5.** Peripheral insulin sensitivity assessed by hyperinsulinemic-

euglycemic clamp.

(A) Plasma insulin concentration during hyperinsulinemic-euglycemic [4 mU / (kg-min)] clamp (n = 9 - 10 per group). (B,C) Plasma glucose concentration and glucose infusion rate time course during clamp, respectively (n = 9 – 10 per group). (D-F) Insulin-stimulated peripheral glucose metabolism (n = 9 - 10 per group), 2deoxyglucose (2DG) uptake in muscle and epididymal adipose tissue (n = 7 - 9 per group), respectively. All data are expressed as mean ± SEM.

**Supporting Figure 6.** Akt phosphorylation at Thr308.

Akt phosphorylation at Thr308 was assessed with the same membrane as Akt phosphorylation at Ser473 assessment. The average expression of control ASO treated basal condition was set as 1. Data are mean ± SEM.

**Supporting Figure 7.** Lysophosphatidic acid acyltransferase acitivity with oleoyl CoA, *de novo* fatty acid synthesis in liver and whole body lipolysis.

(A) Lysophosphatidic acid acyltransferase activity using ^14^C oleoyl CoA (n = 6 per group). (B) *De novo* fatty acid synthesis in liver was calculated based on the incorporation of ^2^H from ^2^H_2_O onto newly synthesized palmitate molecules in hepatic triglyceride-palmitate (n = 7 per group). (C) Whole body lipolysis assessed by glycerol turnover in HFF rats (n = 7 per group). * P<0.05 compared to control ASO treated rats. Data are mean ± SEM.

**Supporting Figure 8.** Adipocyte sizing results.

Adipocyte size was measured using ~10 mg fresh epididymal adipose tissue in HFF rats (n = 11 per group).

**Supporting Materials and Methods**

**Selection of ASOs**

To identify rats pnpla3 inhibitors, rapid-through-put screens were performed in vitro as described previously.^1^ In brief, 80 ASOs were designed to the pnpla3 mRNA sequence, initial screens identified several potent and specific ASOs, all of which targeted a binding site within the coding region of the pnpla3 mRNAs. After extensive dose-response characterization, the most potent ASO from the screen was chosen: ISIS-470521, with the following sequence: 5-GGCAATGAGGTTACCACACA-3. The control ASO, ISIS-141923, has the following sequence: 5-CCTTCCCTGAAGGTTCCTCC-3, and does not have perfect complementarity to any known gene in public databases.

**Hepatic Lipid Metabolites Assay**

After purification, LCCoA, and DAG fractions were separately dissolved in methanol/water (1:1 v/v) and subjected to liquid chromatography / mass spectrometry / mass spectrometry analysis, as previously described.^2^ A turboionspray ionization source was interfaced with an API 3000 tandem mass spectrometer (Applied Biosystems, Foster City, CA). For lysophosphatidic acid (LPA), liver (100 mg) with 1 nmol of C17 LPA was extracted using the method of Bligh and Dyer,^3^ applied to an Oasis HLB column (Waters, Milford, MA) and after washing with water, eluted with methanol. LPAs were measured by electrospray ionization (negative) LC-MS/MS (API 4000 tandem mass spectrometer, Applied Biosystems, Foster City, CA) in conjunction with 2 PerkinElmer 200 Series micro pumps and a 200 Series autosampler (PerkinElmer, Branford, Connecticut). LPA with chain lengths from C6 to C20:4 were monitored for the parent ion (269 to 457) and daughter ion of glycerol-phosphate (m/z 152.9). For phosphatidic acid (PA), the extraction procedure was adapted from the literature methods.^4,5^ Frozen livers (~100 mg) were suspended in precooled mixture of 0.1 ml 0.1 M KH_2_PO_4_ and 1.6 ml CHCl_3_/MeOH (2:1, v:v) containing 1.0 nmol diheptadecanoyl phosphatidic acid as internal standard. The samples were homogenized and transferred into 5 ml glass vial. 0.25 ml 0.1 M KH_2_PO_4_ and 1 ml chloroform were added and vortexed for 1 min, followed by centrifugation for 15 min at 4000 rpm. The organic layer was collected and dried under a nitrogen flow for purification using C18 cartridges (Waters, Milford, MA). C18 cartridges were first conditioned using 2 ml methanol, 2 ml water, and 2 ml methanol. The samples were loaded and phosphatidic acid fraction was eluted with 2.5 ml methanol. The solvent was removed, dried, and reconstituted in 0.25 ml methanol for LC/MS/MS analysis, which was carried out on an API 4000 tandem mass spectrometer (Applied Biosystems, Foster City, CA) with Turbo negative ionization source. A C18 column (2.1 x 50 mm) was used to separate phosphatidic acids with their derivatives using MeOH-water (95:5, v:v) as eluent. Total LCCoA, LPA, PA, and DAG contents were obtained from the sum of individual species, respectively. DAG fractionation into the membrane and the cytosolic lipid droplet compartments was done as previously reported.^6^ Briefly, 50 mg of tissue was homogenized with 400 μl of cold Tris-EDTA-sucrose (TES) buffer (10 mM Tris-Base, pH 7.4, 0.5 mM EDTA, 250 mM sucrose) containing a protease inhibitor cocktail, using a Polytron homogenizer. Homogenates were centrifuged at 100,000 rcf for 1 hour (4 °C). Supernatant (liquid phase, which contains cytosolic lipid droplet) was kept at -20 °C for further use. The pellets were resuspended in 700 μl of TES buffer, which contains plasma membrane, transferred into glass vials and used for the DAG assay as described above.

**Western Blotting**

For PKC membrane translocation assay, protein was compartmentalized into membrane and cytosol compartments. 100 mg of tissue was homogenized in 500 μl buffer A (20 mM Tris-HCl pH 7.4, 1 mM EDTA, 0.25 mM EGTA, 250 mM sucrose and protease inhibitor (Roche Diagnostics, Indianapolis, USA) and centrifuged at 100,000 rcf at 4 °C for 1 hour. The supernatants containing the cytosolic fraction were collected. Pellets were resuspended in 300 μl buffer B (250 mM Tris-HCl pH 7.4, 1 mM EDTA, 0.25 mM EGTA, 2 % Triton X 100 and protease inhibitor cocktail) and centrifuged at 100,000 rcf at 4 °C for 1 hour to obtain the plasma membrane fraction. For protein assays, the plasma membrane fraction was diluted with buffer A 40x to bring the Triton X 100 down to 0.05 %. 50 μg of crude membrane and cytosol protein extracts were used for Western blotting, resolved by SDSPAGE using gradient gel with 15 wells and electroblotted onto polyvinylidene difluoride membrane (DuPont, Boston, MA) using a wet-transfer cell (Bio-Rad, Hercules, CA). The membrane was then blocked for 60 minutes at room temperature in phosphate-buffered saline-Tween (PBS-T:10 mmol/liter NaH_2_PO_4_, 80 mmol/liter Na_2_HPO_4_, 0.145 mol/liter NaCl, and 0.1 % Tween-20, pH 7.4) containing 5 % (w/v) nonfat dried milk, and then incubated overnight with the primary antibodies. Antibodies were diluted as 1:500-1000 in rinsing solution. After further washings, membranes were incubated with horseradish peroxidase-conjugated IgG fraction of goat anti-mouse IgG (Bio-Rad, Hercules, CA) diluted 1: 2000 in TBS-T containing 5 % (w/v) nonfat dried milk for 90 minutes. Both membrane and cytosol proteins were detected with enhanced chemi-luminescense. PKC translocation was expressed as the ratio of arbitrary units of membrane bands over cytosol bands. Membrane band density was corrected by sodium potassium ATPase band density and cytosolic band density was corrected by GAPDH band density.

For whole cell lysate preparation, 100 mg livers were homogenized in 1 ml ice-cold homogenation buffer (20 mM Tris-HCl, pH 7.4, 5 mM EDTA, 0.25 mM EGTA, 10 mM Na_4_P_2_O_7_, 1 %NP-40, 1 mM PMSF, 10 μg/ml aprotinin), centrifuged at 12,000 rcf at 4 °C for 30 minutes. The supernatant was removed into the new tube and protein concentration was determined by the Bradford method (Bio-Rad, Hercules, CA).

For cell fractionation into cytosol, membrane, and lipid droplet, 200 mg of flash frozen liver tissue was homogenized in 800 μl of homogenization buffer C (20 mM Tris-HCl [pH 7.4], 1 mM EDTA, 0.25 mM EGTA, 250 mM sucrose, protease and phosphatase inhibitor cocktail), then 400 μl of 3 % sucrose was layered on top of the homogenate, and samples were centrifuged 100k × g for 1 hour at 4°C. The lipid cake was removed with a 23G1 needle (Becton Dickinson and Company, Franklin Lakes, NJ) and then the cytosol was removed. The pellet was resuspended in Buffer D (20 mM Tris-HCl buffer [pH 7.4], 150 mM NaCl, 50 mM NaF, 1 mM EDTA, 1 mM EGTA, 1 mM dithiothreitol [DTT], phosphatase and protease inhibitor cocktail), homogenized with a 25G7/8 needle, and centrifuged 20,800 × g for 15 minutes at 4 °C. The remaining floating lipid was removed with a 23G1 needle and triton X was added to 2 % v / v. Membrane samples were then passed through a 28G1/2 needle, incubated on ice for 30 minutes, and centrifuged 20,800 × g for 15 minutes at 4°C. The supernatant membrane fraction was removed and saved. Proteins in the lipid fraction were precipitated in acetone in 2 ml tube at -20 °C overnight, centrifuged at 20,800 × g for 30 minutes at 4 °C, the protein pellet was then dried under N_2_ briefly, and resuspended in Buffer B with 1 % triton X v / v. Protein concentration in all three fractions was then determined using the Bradford protein assay method (Bio-Rad, Hercules, CA).

PKCε antibody was purchased from BD Transduction Laboratories (San Diego, CA). GAPDH, Akt, and phosphorylated Akt (Ser473 and Thr308) antibodies were purchased from Cell Signaling Technology, Inc. (Danvers, MA). Sodium potassium ATPase antibodies were purchased from Abcam Inc. (Cambridge, MA). PNPLA3 antibody was purchased from Everest Biotech (Ramona, CA). ADRP antibody was purchased from PROGEN Biotechnik GmbH (Heidelberg, Germany).

**Oil red O staining**

The livers were removed from rats, rinsed in cold phosphate buffered saline, and fixed in 10 % formaldehyde overnight. Then, livers were equilibrated in 5 % sucrose for an hour, 10 % sucrose for an hour, and 20 % sucrose for overnight, and embedded in OCT compound (Sakura Finetek USA, Inc., Torrance, CA) on dry ice. Cryosectioning and staining were done at Research Histology lab in Yale university.

**Biochemical Analysis and Calculations**

Plasma glucose concentrations were measured using a YSI 2700 (YSI Life Sciences, Yellow Springs, Ohio). Plasma insulin was measured by radioimmunoassay kit (Millipore, Billerica, MA). HbA_1C_ was measured in the Geisinger Clinical Laboratory by HPLC. Homeostatic Model Assessment of Insulin Resistance Index (HOMA-IR) was calculated as previously described.^7^ Plasma total cholesterol, triglyceride, and Alanine Aminotransferase (ALT) concentrations were measured on Roche Cobas Mira Plus (Analytical Instruments, LLC, Suite 50 Golden Valley, MN) using the cholesterol, triglyceride, ALT reagent test kit (Raychem, Cliniqa Corporation, San Marcos, CA), respectively. Non-esterified fatty acids were measured on Roche Cobas Mira Plus using the NEFA-HR (2) Color A and B reagent test kit (Wako Chemicals USA, Inc., Richmond, VA). High molecular weight adiponectin was measured with an enzyme-linked immunosorbent assay (ELISA) kit (Millipore Corporation, Billerica, MA).

**Hyperinsulinemic-Euglycemic Clamp Studies**

After 4 weeks of treatment, rats were fasted overnight. The following morning, the clamp study began with a prime (1 mg / kg for 8 min) of 99 % labeled [6,6-^2^H]glucose followed by a continuous infusion at a rate of 0.1 mg / kg per minute for 2 hr to assess the basal glucose turnover. After the basal period, the hyperinsulinemic-euglycemic clamping was conducted for 150 min with a primed/continuous infusion of human insulin (40 mU / kg over 5 min) / [4 mU / (kg-min)] (Novo Nordisk Inc., Princeton, NJ) and a variable infusion of ~20 % dextrose to maintain euglycemia (approximately 100 mg/dl). The dextrose glucose was enriched with [6,6-^2^H]glucose to approximately 2.5 % to match the enrichment in the plasma achieved after the basal period (i.e. “hot-GINF”). A 30 μCi bolus of 2-deoxy-d-[1-^14^C]glucose (American Radiolabeled Chemicals Inc., St. Louis, MO) was injected at 130 min in the clamp to estimate the rate of insulin-stimulated tissue glucose uptake. At the end of the clamp, rats were anesthetized with sodium pentobarbital injection (75 mg / kg), and all tissues were taken within 3 min, frozen immediately using cooled aluminum tongs in liquid N_2_, and stored at -80 °C for the subsequent analysis.

Glucose turnover was calculated at the end of the basal infusion and during the last 40 min of the clamp study. Plasma samples were obtained at 10 minutes intervals and the average of three to four time points’ data was used for the final analysis. To determine the enrichment of [6,6-^2^H]glucose in plasma, 25 μl plasma samples were deproteinized with 25 μl of 0.3N ZnSO_4_ and 25 μl 0.3N Ba(OH)_2_, vortexed, centrifuged at 4000 rpm, 4 °C for 15 min, and supernatants were transferred into another set of tubes, then dried, and derivatized with 1:1 acetic anhydride/pyridine for 15 min at 65 °C to produce the penta-acetate derivative of glucose. The atom percentage of enrichment of glucose_M + 2_ was then measured by gas chromatographic / mass spectrometric (GC/MS) analysis using a Hewlett-Packard 5890 Gas Chromatograph interfaced to a Hewlett-Packard 5971A Mass Selective Detector operating in the electron ionization mode.^8^ Glucose_M + 2_ enrichment was determined from m/z 202 and 200. Rates of basal and insulin-stimulated whole-body glucose turnover were determined as the ratio of the rate of [6,6-^2^H]glucose infusion [mg / (kg-min)] to the atom percentage excess glucose_M + 2_ (%) in the plasma. This rate was corrected by subtraction of the rate of [6,6-^2^H]glucose infusion. Endogenous glucose production during the clamp period was determined by subtraction of the glucose infusion rate from the rate of total glucose appearance. Insulin-stimulated peripheral glucose metabolism was determined by summation of the rate of [6,6-^2^H]glucose infusion, the unlabeled glucose infusion rate, and endogenous glucose production. For the determination of muscle and epididymal adipose tissue [^14^C]2-deoxyglucose-6-phosphate content, tissue samples were homogenized, and the supernatants were subjected to an ion-exchange column to separate [^14^C]2-deoxyglucose-6-phosphate from 2-deoxyglucose. Tissue-specific glucose uptake was calculated from the area under the curve of [^14^C]2-deoxyglucose detected in plasma and the tissue content of [^14^C]2-deoxyglucose-6-phosphate, as previously described.^9^

***In vivo* whole body lipolysis assay**

100 μl plasma was put in the tube. 50 μl of 0.1 mM [2-^13^C]glycerol standard was added, deproteinized with 100 μl of 0.3 N ZnSO_4_ and 100 μl of 0.3 N Ba(OH)_2_, vortexed, centrifuged at 4000 rpm, 4 °C, for 15 minutes. The supernatant was dried, derivatized with 100 μl acetic anhydride and 100 μl pyridine at 65 °C, for 15 minutes. Then, [D-5]glycerol was analyzed for isotope enrichment by GC-MS (EI), by selected ion monitoring, m/z 145-148 (M_0_,M_3_), as previously described.^10^ Atom percentage of enrichment of M_3_ (D_5_ APE) was calculated and corrected with glycerol standard curve. Glycerol turnover rate (Glycerol Ra) was calculated with the following equation: Glycerol Ra = (infusion rate) / [(D5 APE) / 100] - (infusion rate), where infusion rate was 7.5 μmol / kg-min in this time. Finally, whole body lipolysis (μmol / min) was determined by multiplication of body weight.

***In vivo de novo* lipogenesis assay (Assessement of ^2^H labeling in triglyceride-palmitate)**

200 mg liver tissue was homogenized with 4.0 ml of Chloroform: Methanol (1:2) solution. 50 μg of a triglyceride internal standard (Glyceryl triheptadecanoate, Sigma-Aldrich, St. Louis, MO) was added to the homogenate. The homogenate was shaken for 15 minutes at 4 °C, followed by addition of 1.25 ml of chloroform and 1.25 ml of 1N NaCl, and centrifugation at 3500 rpm for 10 minutes. The lower layer was collected, dried under a stream of nitrogen, and re-dissolved in 0.5 ml of chloroform. The lipid extract was separated with a thin layer chromatography plates (Silica Gel 60, GE Healthcare Life Sciences, Piscataway, NJ) developed with hexane: diethylether: acetic acid (80: 20: 1) and the triglyceride spot was scraped into a vial, eluted with diethylether, dried, and derivatized with 0.4 ml of chloroform: methanol (1: 1) solution and 0.1 ml of borontrifluoride (Sigma-Aldrich, St. Louis, MO) at 70 °C for 60 minutes. After cooling, 0.5 ml of water and 1 ml of pentane were added, vortexed and centrifuged at 2000 rpm for 10 minutes. The upper layer (fatty acid layer) was collected, dried under a stream of nitrogen, and redissolved in 120 μl hexane for GC-MS analysis. Then, palmitate was analyzed for isotope enrichment by GC-MS operating in the positive chemical ionization mode (reagent gas: isobutene). Mass isotopomer abundances were analyzed by selected ion monitoring, m/z 271-273 (M_0_-M_2_).

Plasma D_2_O enrichment was also measured by GC-MS. 50 μl of plasma was reacted in a GC vial with calcium carbide (Sigma-Aldrich, St. Louis, MO) to produce acetylene. Head-space acetylene was analyzed for isotope enrichment by selective ion monitoring of m/z 26 to 28 using a Hewlett-Packard 6890 Gas Chromatograph interfaced to a Hewlett-Packard 5973A Mass Selective Detector operating in the electron ionization mode.

*De novo* lipogenesis (%), newly synthesized palmitate in the hepatic triglyceride-palmitate was calculated as previously described^11^ based on the incorporation of ^2^H from ^2^H_2_O onto newly synthesized palmitate molecules. First, atom percentage of enrichment of M_1_ (and M_2_), m1 (and m2), was calculated with the equation: m1 = 100 × (M_1_/M_0_ - natural M_1_/M_0_) / [1 + (M_1_/M_0_ - natural M_1_/M_0_)]. Then, molar enrichment (ME) was calculated with the equation: ME = m1 + 2 × m2. Finally, the newly synthesized fraction (F) was calculated with the equation: F = ME / (N × p), where N, the number of exchangeable hydrogens, has been reported as 22^11,12^ and p = plasma D_2_O atom percentage of enrichment. *De novo* synthesis (%) was determined by multiplication of 100 to F.

**Adipocyte size and number calculation**
Cell-size distribution was described via a mathematical model in which a formula with seven cell-size parameters was fit to the cell-size distribution, as previously described.^13^ For each participant, analysis of adipose cell-size distribution from Multisizer graphs entailed identification of the nadir, which was defined as the low point (in frequency) between the two cell populations; the number of adipose cells above and below this point was calculated by the Multisizer software, and expressed as the ‘% above’ (% large cells) and ‘% below’ (% small cells) the nadir, as well as the ratio of small to large cells the midpoint of the flat region between the two populations was designated the nadir.^13^ All cells beneath the Gaussian curve were considered as the fraction of large cells (fraclarge) and the ‘peak diameter’ of the large adipose cells was defined as the mean diameter at which the frequency of the large cell population reached a maximum.^13^ A secondary endpoint, the number of subcutaneous adipose cells, was estimated by the following formula: cell number = volume of subcutaneous abdominal adipose tissue/weighted volume per cell. Volume of adipose tissue was obtained from MRI scans and average volume per cell was calculated as the weighted volume based on the relative number of cells per volume bin in the cell-volume histogram generated by the Multisizer software. We used the following formula: average volume per cell = ∑ 4/3 π (di/2)3 pi  (that is, the sum of the volumes corresponding to each bin times the relative frequency (p) of that bin (i).^14^

**Supporting References**

1. Watts LM, Manchem VP, Leedom TA, Rivard AL, McKay RA, Bao D, Neroladakis T, et al. Reduction of hepatic and adipose tissue glucocorticoid receptor expression with antisense oligonucleotides improves hyperglycemia and hyperlipidemia in diabetic rodents without causing systemic glucocorticoid antagonism. Diabetes 2005;54:1846-1853.

2. Kumashiro N, Erion DM, Zhang D, Kahn M, Beddow SA, Chu X, Still CD, et al. Cellular mechanism of insulin resistance in nonalcoholic fatty liver disease. Proc Natl Acad Sci U S A 2011;108:16381-16385.

3. Bligh EG, Dyer WJ. A rapid method of total lipid extraction and purification. Can J Biochem Physiol 1959;37:911-917.

4. Lee JY, Min HK, Moon MH. Simultaneous profiling of lysophospholipids and phospholipids from human plasma by nanoflow liquid chromatography-tandem mass spectrometry. Anal Bioanal Chem 2011;400:2953-2961.

5. Holland WL, Stauter EC, Stith BJ. Quantification of phosphatidic acid and lysophosphatidic acid by HPLC with evaporative light-scattering detection. J Lipid Res 2003;44:854-858.

6. Bogan JS, McKee AE, Lodish HF. Insulin-responsive compartments containing GLUT4 in 3T3-L1 and CHO cells: regulation by amino acid concentrations. Mol Cell Biol 2001;21:4785-4806.

7. Matthews DR, Hosker JP, Rudenski AS, Naylor BA, Treacher DF, Turner RC. Homeostasis model assessment: insulin resistance and beta-cell function from fasting plasma glucose and insulin concentrations in man. Diabetologia 1985;28:412-419.

8. Hundal RS, Petersen KF, Mayerson AB, Randhawa PS, Inzucchi S, Shoelson SE, Shulman GI. Mechanism by which high-dose aspirin improves glucose metabolism in type 2 diabetes. J Clin Invest 2002;109:1321-1326.

9. Youn JH, Buchanan TA. Fasting does not impair insulin-stimulated glucose uptake but alters intracellular glucose metabolism in conscious rats. Diabetes 1993;42:757-763.

10. Weiss R, Taksali SE, Dufour S, Yeckel CW, Papademetris X, Cline G, Tamborlane WV, et al. The "obese insulin-sensitive" adolescent: importance of adiponectin and lipid partitioning. J Clin Endocrinol Metab 2005;90:3731-3737.

11. Lee WN, Bassilian S, Ajie HO, Schoeller DA, Edmond J, Bergner EA, Byerley LO. In vivo measurement of fatty acids and cholesterol synthesis using D2O and mass isotopomer analysis. Am J Physiol 1994;266:E699-708.

12. Wadke M, Brunengraber H, Lowenstein JM, Dolhun JJ, Arsenault GP. Fatty acid synthesis by liver perfused with deuterated and tritiated water. Biochemistry 1973;12:2619-2624.

13. McLaughlin T, Sherman A, Tsao P, Gonzalez O, Yee G, Lamendola C, Reaven GM, et al. Enhanced proportion of small adipose cells in insulin-resistant vs insulin-sensitive obese individuals implicates impaired adipogenesis. Diabetologia 2007;50:1707-1715.

14. Jo J, Gavrilova O, Pack S, Jou W, Mullen S, Sumner AE, Cushman SW, et al. Hypertrophy and/or Hyperplasia: Dynamics of Adipose Tissue Growth. PLoS Comput Biol 2009;5:e1000324.
