# Supplementary material for: Role of Patatin-Like Phospholipase Domain-Containing 3 on Lipid-Induced Hepatic Steatosis and Insulin Resistance in Rats
Source: Hepatology. 2013 Jan 25;57(5):1763–72. doi: 10.1002/hep.26170 (PMC3597437; doi:10.1002/hep.26170)
Supplement: Supplementary file 10 [file hep0057-1763-sd10.doc]

**Supporting Table 1. Plasma data.**

|  | Regular chow | | High fat diet | |
| --- | --- | --- | --- | --- |
|  | Control ASO | Pnpla3 ASO | Control ASO | Pnpla3 ASO |
| ALT (IU / L) | 36.2 ± 6.9 | 35.0 ± 1.2 | 36.3 ± 4.2 | 38.4 ± 0.8 |
| Total cholesterol (mmol / L) | 2.95 ± 0.12 | 3.21 ± 0.05 | 3.06 ± 0.19 | 2.59 ± 0.16 |
| Triglyceride (mmol / L) | 0.22 ± 0.01 | 0.23 ± 0.02 | 0.21 ± 0.02 | 0.19 ± 0.02 |
| NEFA (mmol / L) | 1.03 ± 0.06 | 1.04 ± 0.09 | 1.00 ± 0.06 | 0.81 ± 0.05 |
| Adiponectin (μg / mL) | 1.94 ± 0.21 | 2.44 ± 0.33 | 1.93 ± 0.15 | 2.06 ± 0.29 |

ALT; Alanine Aminotransferase, NEFA; nonesterified plasma fatty acid

Data are means ± SEM.
