# Supplementary material for: Role of Patatin-Like Phospholipase Domain-Containing 3 on Lipid-Induced Hepatic Steatosis and Insulin Resistance in Rats
Source: Hepatology. 2013 Jan 25;57(5):1763–72. doi: 10.1002/hep.26170 (PMC3597437; doi:10.1002/hep.26170)
Supplement: Supplementary file 11 [file hep0057-1763-sd11.doc]

**Supporting Table 2. DAG species analysis.**

| Species | Control ASO | Pnpla3 ASO |
| --- | --- | --- |
| C18:2, C18:2 | . .189 ± 43 | .59 ± 11* |
| C20:4, C20:5 | . .166 ± 16 | .141 ± 13 |
| C18:1, C18:2 | . .158 ± 22 | .95 ± 10* |
| C16, C18:2 | . .146 ± 28 | .60 ± 5* |
| C18:1, C18:1 | .57 ± 9 | .34 ± 2* |
| C18:1, C16 | .25 ± 6 | .10 ± 1* |
| C18:2, C18 | .14 ± 3 | 6.2 ± 0.7* |
| C18, C20:4 | 5.8 ± 0.5 | 4.9 ± 0.4 |
| C16, C16 | 5.3 ± 1.0 | 3.3 ± 0.4 |
| C18, C18 | 4.7 ± 0.3 | 3.8 ± 0.3 |
| C16, C20:4 | 4.2 ± 0.3 | 2.9 ± 0.2** |
| C18:1, C18 | 2.8 ± 0.6 | 1.5 ± 0.2 |
| C18, C16 | 2.7 ± 0.4 | 1.7 ± 0.2 |

Membrane DAGs were assessed by individual species in HFF overnight fasted rats. Data are means ± SEM [nmol / (g-liver)]. * P<0.05, ** P<0.01 compared with control ASO treated rats.
