# Supplementary material for: Role of Patatin-Like Phospholipase Domain-Containing 3 on Lipid-Induced Hepatic Steatosis and Insulin Resistance in Rats
Source: Hepatology. 2013 Jan 25;57(5):1763–72. doi: 10.1002/hep.26170 (PMC3597437; doi:10.1002/hep.26170)
Supplement: Supplementary file 12 [file hep0057-1763-sd12.doc]

**Supporting** **Table 3. Characteristics of participants.**

|  | Total | Wild | Hetero | Homo |
| --- | --- | --- | --- | --- |
| Number | 35 | 25 | 3 | 7 |
| Female / Male | 28 / 7 | .19 / 6 | 3 / 0 | 6 / 1 |
| Age (y) | 42.1 ± 2.3 | 40.0 ± 2.4 | 47.3 ± 12.4 | 47.4 ± 5.9 |
| Body-mass index (kg / m^2^) | 48.2 ± 1.5 | 48.9 ± 1.9 | 57.8 ± 1.7 | 42.0 ± 1.5 |
| Fasting plasma glucose (mg / dL) | 97.8 ± 3.0 | 100.2 ± 3.7 | 95.0 ± 12.0 | 92.8 ± 5.7 |
| Fasting plasma insulin (μU / mL) | 22.0 ± 1.6 | 22.6 ± 2.2 | 28.5 ± 0.9 | 18.7 ± 2.5 |
| HbA_1C_ (%) | .5.5 ± 0.1 | 5.5 ± 0.1 | .5.7 ± 0.2 | .5.6 ± 0.2 |
| HOMA-IR [(mg / dl)*(μU / mL)] | .4.7 ± 0.4 | 4.8 ± 0.5 | .5.6 ± 1.3 | .4.0 ± 0.6 |
| Alanine aminotransferase (IU / L) | 28.1 ± 2.4 | 30.5 ± 3.2 | 30.0 ± 5.7 | 20.4 ± 2.5 |
| Aspartate aminotransferase (IU / L) | 24.6 ± 1.3 | 25.2 ± 1.8 | 26.0 ± 1.7 | 22.3 ± 1.8 |
| LDL cholesterol (mmol / L) | 2.67 ± 0.18 | 2.86 ± 0.23 | 1.99 ± 0.43 | 2.47 ± 0.30 |
| HDL cholesterol (mmol / L) | 1.12 ± 0.04 | 1.09 ± 0.06 | 1.11 ± 0.09 | 1.22 ± 0.06 |
| Triglyceride (mmol / L) | 1.72 ± 0.20 | 1.86 ± 0.25 | 0.95 ± 0.17 | 1.68 ± 0.45 |

Data are expressed as mean ± SEM.

HbA_1C_, Hemoglobin A_1C_; HOMA-IR, Homeostatic Model Assessment of Insulin Resistance Index; LDL, Low-density lipoprotein; HDL, High-density lipoprotein; Hetero and Homo are heterozygotes and homozygotes for the 148M mutant allele, respectively.
