# Supplementary material for: Role of Patatin-Like Phospholipase Domain-Containing 3 on Lipid-Induced Hepatic Steatosis and Insulin Resistance in Rats
Source: Hepatology. 2013 Jan 25;57(5):1763–72. doi: 10.1002/hep.26170 (PMC3597437; doi:10.1002/hep.26170)
Supplement: Supplementary file 13 [file hep0057-1763-sd13.doc]

**Supporting Table 4. Primer sequences for RT-PCR.**

|  | Forward | Reverse |
| --- | --- | --- |
| PNPLA3 (H) | AAGTTCCTCCGACAGGGTCTCT | GCCTATTTTGCCGGAGATGA |
| Tbp (H) | CACAGTGAATCTTGGTTGTAAACTTGA | AAACCGCTTGGGATTATATTCG |
| Tbp (R) | GGACTCCTGTCTCCCCTACC | CTCAGTGCAGAGGAGGGAAC |
| SREBP1c (R) | GGGAACACCCAGCAGACGCC | GTGCGCTTCTCACCACGGCT |
| ACC1 (R) | GTTCGAAGAGCTTATATCGCCTATG | GGGCAGCATGAACTGAAATTC |
| FAS (R) | CCAGGAACTGAACGGCATTAC | GATTTGGTGGAGCCAATTAACAA |
| mGPAT (R) | CGCTGAAATGGAAGGAGAG | ACCCAGAGATGGGATACTGG |
| AGPAT1 (R) | CCCACACAGCCCTACGTTGT | CTGGCAGTACCTCCATCATTCC |
| AGPAT2 (R) | AGGAATGGGCAACGCATTAC | GCCTGGTTCCTGAATGTTTGTC |
| AGPAT3 (R) | CGGAGGCCGTGGACTCT | GACGCCCAGGACGAAGCT |
| AGPAT4 (R) | GCCCAGCCTCAAGCATCA | ACATCTCGCAAGCACTTCACA |
| AGPAT5 (R) | CAGCATCCTGTCTGTCCTAGCA | AAACGCAGTGCCAAAAGCA |
| AGPAT6 (R) | TCCCTGACCGTCCTCTTCAC | CGGATACCAAAGGACACTCCAA |
| AGPAT9 (R) | GAACAACAGAAGACTTACAGCAAAATG | CATGATTGTCCACAGTGCTTGA |
| PAP (R) | GAGGCCAGGCTGTCCTTCTA | GAGCGCCAGGAACAACATG |
| DGAT2 (R) | AGGCCTTGATGGTTTCTATCCA | GCTGCCCTTCCCCAATTAAC |
| ATGL (R) | TCTCGGCTCACCCGGCTACC | GGGCTCCTCTGAGTGGGGCA |
| PPARα (R) | CAGGAGAGCAGGGATTTGCA | CCTAGGCTCAGCCCTCTTCA |
| CPT1 (R) | GGTTCAAGAATGGCATCATCACT | ATCACACCCACCACCACGATA |
| Pnpla3 (R) | TCCAAAGACGAAGTGGTGGAT | GGAGGGATTAGGCCAGAGAAGA |

PNPLA3, Patatin–like phospholipase domain-containing 3; Tbp, TATA box binding protein; SREBP, sterol regulatory element binding transcription factor; ACC, acetyl-CoA carboxylase; FAS, fatty acid synthase; mGPAT, mitochondrial acyl-CoA:glycerol-sn-3-phosphate acyltransferase; AGPAT, acyl-CoA:1-acylglycerol-sn-3-phosphate acyltransferase; AGPAT1, 2, 3, 4, 5, 6, and 9 are transcript variants of AGPAT; PAP, phosphatidic acid phosphatase; DGAT, acyl-CoA:diacylglycerol acyltransferase; ATGL, adipocyte triglyceride lipase; PPARα, peroxisome proliferator activated receptor alpha; CPT, carnitine palmitoyl transferase. (H); primers for humans, (R); primers for rats.
